# Loss of NF1 Accelerates Uveal and Intradermal Melanoma Tumorigenesis, and Oncogenic GNAQ Transforms Schwann Cells

**DOI:** 10.1158/2767-9764.CRC-24-0386

**Published:** 2025-02-03

**Authors:** Anne Nathalie Longakit, Oscar Urtatiz, Amy Luty, Christina Zhang, Chloe Hess, Alyssa Yoo, Hannah Bourget, Catherine D. Van Raamsdonk

**Affiliations:** Department of Medical Genetics, Life Sciences Institute, University of British Columbia, Vancouver, Canada.

## Abstract

*NF1* encodes the multifunctional tumor suppressor protein, neurofibromin, which is best known for its causative role in neurofibromatosis type 1 and in regulating MAPK signaling. Neurofibromin, in a context-specific manner, is involved in various tumorigenic processes, including those in melanocytes. This study investigated whether *NF1* loss can collaborate with oncogenic GNAQ to promote melanoma in the dermis or eyes, in which the Gα_q_ pathway is almost always activated. We found that heterozygous 17q11.2 loss that includes the *NF1* locus is a recurrent phenomenon in human intradermal and uveal melanomas described in the literature. We studied the effects of *Nf1* haploinsufficiency in mice expressing oncogenic *GNAQ*^Q209L^ in melanocytes and Schwann cells of peripheral nerves using the *Plp1-creERT* transgene with tamoxifen at 5 weeks of age. *Nf1* haploinsufficiency accelerated melanoma formation and/or growth. RNA sequencing analysis found significant pathways related to cAMP signaling and myogenesis. In addition, 20% of the differentially expressed genes were homologous to genes for which expression correlates with prognosis in human uveal melanoma. Unexpectedly, we found that *GNAQ*^*Q209L*^ alone was sufficient to drive cutaneous nerve sheath tumors, with one *GNAQ*^*Q209L*^-expressing *Nf1* haploinsufficient mouse also developing a plexiform variant. These tumors strongly resembled neurofibromas. We searched the cBioPortal for Cancer Genomics database and found plexiform neurofibromas with the *GNAQ*^*T96S*^ hotspot mutation. The *Plp1-creERT*; *GNAQ*^*Q209L*^ model with tamoxifen at 5 weeks may be useful as a preclinical model for neurofibroma. Our studies emphasize the importance of *GNAQ* and *NF1* in regulating neural crest–derived cells existing in a dermal-like environment.

**Significance:**

These results indicate that *NF1* loss in intradermal and uveal melanomas is a potentially significant finding. They emphasize the importance of neurofibromin in cAMP signaling. They show for the first time that oncogenic GNAQ can transform Schwann cells in mice. The *Plp1-creERT* transgene with tamoxifen given at 5 weeks may be a particularly good strategy for modeling cutaneous neurofibroma and plexiform neurofibroma.

## Introduction

MAPK pathway activation is one of the key events in melanomagenesis, as well as in many other cancers (1). Somatic mutations that activate the MAPK pathway in cutaneous melanoma include oncogenic mutations at specific hotspots in *NRAS* and *BRAF* (2, 3) and tumor suppressor mutations in negative regulators, such as *RASA2* and *NF1* (4, 5). The heterotrimeric G protein α subunits, Gα_q_ and Gα_11_, also participate in MAPK activation, and oncogenic mutations in these two genes are very frequent in melanocytic lesions in the dermis, meninges of the central nervous system (CNS), and the uveal tract of the eye (6–9). The oncogenic mutations in *GNAQ* and *GNA11* at Q209 or R183 cause constitutive activity, preventing Gα_q_ and Gα_11_ from performing GTP hydrolysis and returning to an inactive GDP-bound state. Gα_q_ and Gα_11_ activate phospholipase C β 4, which stimulates protein kinase C by way of the second messenger, diacylglycerol (DAG). Protein kinase C (PKC) activates RASGRP3 feeding into the MAPK pathway (10).

Although oncogenic MAPK mutations are mutually exclusive with each other (11), tumor suppressor *NF1* mutations have more of a cooperative or additive effect. For example, nearly two thirds of *NF1*-mutant cutaneous melanomas carry a second MAPK gene hit (5, 12). *NF1* mutations are present in 5% of *BRAF-*, 13% of *NRAS-*, and 50% of *RASA2*-mutant cutaneous melanomas. When *NF1* mutations occur in combination with another MAPK hit, the *NF1* mutations are more likely to be heterozygous, presumably acting through haploinsufficiency (4). When *NF1* is the only mutated MAPK gene, both alleles of *NF1* are typically mutant. This occurs by either compound heterozygous mutations or one *NF1* focal mutation plus loss of heterozygosity. Zeng and colleagues (13) and others have suggested that MAPK pathway activation in melanoma is not an “all or nothing” phenomenon.

In this study, we investigated a possible cooperative role of *NF1* loss in the context of *GNAQ*-mutant melanoma. *NF1*, which is located at 17q11.2, encodes the very large neurofibromin protein (14). Neurofibromin is a multifunctional protein that, in context-specific ways, regulates MAPK, PI3K/AKT/mTOR, Rho/ROCK/LIMK2/cofilin, PKA-Ena/VASP, and cAMP/PKA signaling (reviewed in ref. 15). This affects various cellular processes related to tumorigenesis, including proliferation, migration, cytoskeletal dynamics, and apoptosis.

In addition, germline heterozygous loss-of-function mutations in *NF1* cause neurofibromatosis type 1. In addition to many other symptoms, individuals with neurofibromatosis develop neurofibromas when there is a second, somatic hit in *NF1* in Schwann cells. Neurofibromas are complex tumors containing Schwann cells, fibroblasts, perineural cells, and mast cells in a variably myxoid background. People with neurofibromatosis type 1 also have pigmentation alterations, such as generalized and subtle skin hyperpigmentation and flat, circumscribed café au lait macules (16). There are case reports of uveal melanoma occurring in patients with neurofibromatosis type 1 (17–19), and it has been estimated that twice as many cases of uveal melanoma have been reported than would be expected by chance (20). Another study found that *NF1* expression was downregulated in uveal melanoma (21).

We previously studied conditional and constitutive knockout *Nf1* mutations in mice and found that there is tail skin hyperpigmentation (22, 23). Histology of the mouse tail skin showed that the epidermis was darker, similar to the generalized skin hyperpigmentation in human patients with *NF1* mutations. In addition, the dermis was hyperpigmented in the *Nf1*-mutant mice, suggesting that *NF1* regulates melanocytes outside the epidermis (22).

To determine whether *NF1* loss plays a role in GNAQ-driven melanoma, such as forms in the dermis or eye, we surveyed the published literature and The Cancer Genome Atlas (TCGA) uveal melanoma dataset (UVM) to see whether there were copy-number changes in *NF1* reported in intradermal melanocytic lesions (“blue nevus” types) or uveal melanoma. We found that 14% of malignant, but not benign, intradermal lesions exhibited copy-number loss that included the *NF1* gene. There were also two cases of uveal melanoma with *NF1* copy-number loss among the 80 TCGA-UVM samples, and these cases had some other intriguing molecular features. Next, to test the effect of *Nf1* loss in a model system, we forced the expression of oncogenic *GNAQ*^*Q209L*^ using *Plp1-creERT* with tamoxifen at 5 weeks of age and studied the effects of knocking out one copy of *Nf1*. We found that *Nf1* heterozygous loss accelerated the development of intradermal and uveal melanomas. We investigated the transcriptional changes that accompany *Nf1* loss in the context of intradermal and uveal melanomas and found evidence for upregulation of cAMP signaling and downregulation of myogenesis gene expression, respectively. Lastly, we unexpectedly discovered that *GNAQ*^*Q209L*^ expression in *Plp1*-expressing cells can drive the formation of neoplasms similar to cutaneous neurofibromas even without *Nf1* loss. One *GNAQ*^*Q209L*^-expressing *Nf1* haploinsufficient mouse also developed a large plexiform variant in the armpit. We searched the cBioPortal for Cancer Genomics database and found plexiform neurofibromas with the *GNAQ*^*T96S*^ hotspot mutation. This corroborates our findings that oncogenic GNAQ can drive neurofibroma as well as melanoma.

## Materials and Methods

### Mice

The research described in this article was conducted under the approval of the University of British Columbia (UBC) Animal Care Committee (UBC animal care protocol number A19-0152, C.V.R). *Nf1*^*flox*^ (*Nf1*^*tm1Par*^), *Plp1-cre ERT* [*Tg(Plp1-cre/ERT)3Pop*], *Rosa26-fs-GNAQ*^*Q209L*^ [*Gt(ROSA)26Sor*^tm1(GNAQ*)Cvr^], and *Rosa26-fs*-*tdTomato* [*Gt(ROSA)26Sor*^*tm14(CAG-tdTomato)Hze*^] mice were genotyped as previously described (24–28). Each allele was backcrossed to the C3HeB/FeJ genetic background for at least four generations before use. DNA from ear notches was isolated using DNeasy Blood and Tissue Kit (Qiagen) and amplified using PCR with HotStar Taq (Qiagen). Mice in the study were bred in two sequential cohorts. The first cohort established the development of tumors, and the second cohort was used to increase numbers. There were roughly equal numbers of *Nf1*^*flox/*^*+* and *Nf1 +/+* mice expressing *GNAQ*^*Q209L*^ in each cohort.

### Tamoxifen

Tamoxifen (Sigma, T5648) was dissolved in a corn oil/ethanol (10:1) mixture at a concentration of 10 mg/mL by gentle inversion at 37°C for 30 minutes and then stored at 4°C for up to 1 week. At 5 weeks of age, sterile filtered tamoxifen (1 mg in 0.1 mL) was administered through intraperitoneal injection twice per day (every 12 hours) for 3 consecutive days.

### Animal monitoring

Animals were monitored to calculate a clinical health score in each of the following categories: weight, activity level, appearance, posture/gait, and tumor size. Mice were euthanized when an externally visible tumor reached >0.5 cm in diameter or there was another health concern affecting animal welfare (piloerection, hunching, excessive scratching, and severely thickened ear skin without a single tumor >0.5 cm.)

### Histology

Embedding and hematoxylin and eosin (H&E) staining were performed by Wax-it Histology Services (Vancouver, B.C.). Skin samples and tumors were fixed in 10% buffered formalin overnight at room temperature with gentle shaking. Then the samples were dehydrated, cleared, embedded in paraffin, and sectioned at 5 μm before staining using the standard H&E technique. Eye samples were prepared in the same way, except fixation was performed in the Davidson fixative for 3 hours, followed by 10% formalin for 1 hour at 4°C. For visualization of tomato fluorescence, eyes were fixed in 10% buffered formalin overnight at 4°C, taken through a sucrose gradient, embedded in optimal cutting temperature compound (O.C.T.), and sectioned at 10 μm. Sections were washed in 1× PBS and counterstained with 4',6-diamidino-2-phenylindole (DAPI). Images were collected using an Axio Scan.Z1 slide scanner (Zeiss).

### Cutaneous tumor measurement

At euthanasia, the length (A) and width (B) of each cutaneous tumor were measured from above using a ruler. The tumor was embedded, and an H&E-stained section that showed the full thickness of the tumor was used to measure tumor depth (C). Tumors were ellipsoid in shape, and so the following equation was used to determine volume: 4/3 × π × A × B × C.

### IHC

Our complete protocol for IHC on pigmented tissue will be submitted to *Bio-protocol* (https://bio-protocol.org/en). Briefly, 5-μm paraffin sections were first dewaxed and rehydrated into 1× PBS. Antigen retrieval was performed by incubating the slides in 0.6 L of 98°C citrate buffer pH 6.0 (Vector, H-3300) for 10 minutes, followed by removal from the heat source and cooling for 40 minutes at room temperature. Next, the sections were washed and then placed in a solution of 10% H_2_O_2_ in 1× PBS, which was then heated to 60°C in an oven and held until all pigment was removed (2.5 hours). Sections were washed again, blocked in 5% normal serum in 1× PBS plus Triton 0.3% for 1 hour at room temperature, and then incubated with the primary antibody (anti-S100b diluted 1:1,000, Abcam, ab5264; or anti-RPE65 diluted 1:250, Thermo Fisher Scientific, MA1-16578; or anti-CD34 diluted 1:200, Abcam, ab8158) diluted in blocking solution for 1.5 hours at room temperature. Primary antibody was detected using an Elite ABC kit for horseradish peroxidase (HRP; Vector) using 3,3'-diaminobenzidine (DAB) with nickel (Vector, SK-4100) as directed. Some slides were then counterstained with hematoxylin. Stained slides were scanned for digital imaging (Panoramic 250 Flash III whole-slide scanner, 3DHISTECH). The sparse pigment tumors were incubated with a myelin basic protein (Mbp) antibody (Abcam, ab218011) as above but without the bleaching step, which was not compatible with this antibody.

### Measurement of spinal and CNS lesions

To quantify the surface area of the spine or brain that was affected, tissues were photographed under constant conditions alongside a ruler. In ImageJ, each cross-section image was transformed into a binary image. A freehand region of interest was drawn (around the lesion), and the threshold function was used to set threshold limits that covered the pigmented part of this area. The measure function was then used to compute the affected area in relative units. These were converted to mm^2^ using the ruler in each photo.

### Measurement of uveal melanomas

We computed the pigmented uveal tract thickness by analyzing eye H&E cross-sections using ImageJ. Images were taken from the center of the eye (as indicated by the presence of the optic nerve). A freehand region of interest was drawn, and the threshold function was used to set threshold limits that covered the pigmented tissue in the eye. The measure function was then used to compute this area in relative units. These were converted to mm^2^ using the scale bar in each image.

### RNA sequencing and differential expression analyses

All *GNAQ*^*Q209L*^-expressing mice in the second cohort of breeding were used for RNA sequencing (RNA-seq). The second cohort of mice can be identified in Supplementary Table S1A as mice with ID numbers that start with the number six. The tumor type collected from each mouse is also noted. Dermal tumor tissue was collected for RNA extraction when each mouse was euthanized for a dermal tumor. At the same time, one eye was removed from each mouse for RNA-seq and the other eye was embedded for histology. For dermal tumors, overlying epidermis and fat were removed before placing a small piece of the tumor into TRIzol (Life Technologies) for immediate homogenization. For eyes, the globes were removed using curved forceps and placed on a chilled Petri dish on ice. Each eye was pulled open using fine forceps, and pieces of thickened pigmented tissue were collected into TRIzol for immediate homogenization. RNA was then isolated according to the manufacturer’s protocol. Further steps were performed by the BRC sequencing core at the University of British Columbia. Quality control of the RNA samples was performed using the Agilent 2100 Bioanalyzer. Samples were then prepped according to the standard protocol for NEBNext Ultra II Stranded mRNA (New England Biolabs). All samples had an RNA integrity number greater than or equal to 7.9. The samples were all poly(A)-selected. RNA-seq was performed on the Illumina NextSeq 918 500 with paired-end 43 bp × 43 bp reads. The library consisted of ∼20 to 25 million reads in total per sample. Illumina’s bcl2fastq2 was used for demultiplexing the sequencing data, and these reads were then aligned to the reference genome of *Mus musculus* using STAR aligner. The FASTQ files are available at the Sequence Read Archive database under project PRJNA1100739 (https://www.ncbi.nlm.nih.gov/sra/PRJNA1100739). The aligned read counts were used as input files for differential expression (DE) analysis using DESeq2 on R 4.0.3 following the “rnaseqGene” Bioconductor package.

### Statistical analysis

Analyses for gene ontology (GO) were performed using Enrichr. Statistical analyses of mouse phenotypes and survival were performed using Prism. TCGA-UVM Kaplan–Meier survival curves were produced using Survival Genie (29).

### Data availability

Sequencing data were generated by the authors and the UBC BRC sequencing core facility and were deposited in the Sequence Read Archive repository at https://www.ncbi.nlm.nih.gov/sra/PRJNA1100739. All other raw data were generated by the authors and are available upon request of the corresponding author.

## Results

### Review of 17q11.2 copy-number changes in intradermal melanocytic lesions

We surveyed the literature for studies that molecularly examined intradermal nevi and melanomas, which are commonly called blue nevus–type lesions. Few studies had sequenced the *NF1* locus in these types of lesions. One case, a cellular blue nevus, was mutant for *NF1* (*NF1*^*S856R*^; ref. 30). There were, however, 115 cases with genome-wide copy-number analysis (Table 1; refs. 31–37). These cases were described as benign, intermediate/ambiguous, or malignant in their corresponding publications (color coded in Table 1). The copy number was assessed using array comparative genomic hybridization, molecular inversion probe technology, or next-generation sequencing. Copy-number alterations (CNA) were absent in the 86 benign or intermediate cases. Of the 29 malignant cases, 4 (14%) exhibited partial loss of chromosome 17, which included 17q11.2 (31–33). This suggested that haploinsufficiency of *NF1* could play a role in the progression of blue nevus–type lesions.

**Table 1:** 115 intradermal melanocytic lesions with available 17q11.2 copy-number status

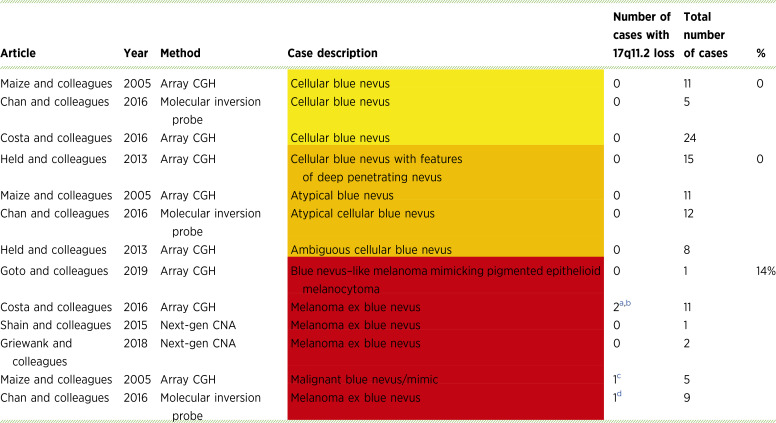

Abbreviations: CGH, comparative genomic hybridization; Next-gen, next generation.

Yellow cells indicate benign lesions, orange cells indicate intermediate-grade lesions, and red cells indicate malignant melanoma.

a dim(17) (q11-q22).

b dim(17) (q11-q22).

c dim(17) (pter-q21).

d dim(17) (q11.2-q21.31).

### Review of 17q11.2 copy-number changes in the TCGA-UVM dataset

We next examined *NF1* in uveal melanoma. Uveal melanoma arises from melanocytes located in the uveal tract of the eye (38). There are 80 cases of primary uveal melanoma in the TCGA-UVM dataset (https://portal.gdc.cancer.gov; ref. 39). We examined these cases for *NF1* mutation status and CNAs. No somatic point mutations in *NF1* were found in uveal melanoma. However, two cases carried a heterozygous, partial loss of chromosome 17 that included the *NF1* gene. The copy-number loss spanned 17q11.2-17q25.2 (*TWF1P1* to *JMJD6*) in case TCGA-VD-AA8M and 17p13.3-q21.32 (*DOC2B* to *HOXB2*) in case TCGA-VD-AA8Q. Both cases carried a typical glutamine substitution at Q209 in *GNAQ*. Strikingly, TCGA-VD-AA8Q carried a *RASA2* mutation (*Rasa2*^*K81Q*^). This alteration was rated with a SIFT impact score of 0.03 (deleterious) and a Polyphen score of 0.977 (probably damaging). Recurrent mutations at neighboring residue S82 have been previously reported in sun-exposed melanomas (5). The K81Q mutation was the only *RASA2* mutation present in the TCGA-UVM dataset, so its co-occurrence with *NF1* copy-number loss is quite interesting, given the previously observed co-occurrence of *RASA2* and *NF1* mutations in cutaneous melanomas (12). TCGA-VD-AA8Q was reported to end in death by metastatic disease. Both cases lacked mutations in the common uveal melanoma tumor suppressors, *SF3B1*, *BAP1*, and *EIF1AX*. We also note that there are six other cases with copy-number gain (3× or 4×) that included the *NF1* locus, with unknown significance.

### Two dermal tumor types found in *GNAQ*^Q209L^-expressing mice

We next induced heterozygous loss of *Nf1* in our *GNAQ*^*Q209L*^-expressing mouse model as an experimental system to test the role of *Nf1* in nonepithelial melanoma. To drive oncogenic *GNAQ*^*Q209L*^ expression in mice, we used the previously described *Rosa26-floxed stop-GNAQ*^*Q209L*^ (“*R26-fs-GNAQ*^*Q209L*^”) allele (26). In this allele, constitutively active human *GNAQ*^*Q209L*^ was knocked into the ubiquitously expressed *Rosa26* locus, preceded by a *loxP* flanked stop cassette that prevents transcription. In cells that express Cre recombinase, the two *loxP* sites are recombined and the intervening stop cassette is deleted, allowing *GNAQ*^*Q209L*^ to be expressed. To knockout one copy of *Nf1*, we used the conditional *Nf1*^*tm1Par*^ (“*Nf1*^*flox*^”) mice (27). These alleles were combined with the widely used tamoxifen-inducible *Plp1-creERT* transgene [*Tg(Plp1-cre/ERT)3Pop*]. This line is expressed in melanocytes and peripheral nerve sheath cells (Schwann cells) and was of interest to us due to its previous connections with *Nf1* and tumorigenesis (22, 40–44). All mice were of an inbred C3HeB/FeJ genetic background.

We induced CreERT activity 5 weeks after birth using twice-daily intraperitoneal injections of tamoxifen for 3 days. We injected *Plp1-creERT* mice that carried just *R26-fs-GNAQ*^*Q209L*^/+ (*n* = 14 mice) or *R26-fs-GNAQ*^*Q209L*^/+ and *Nf1*^*flox*^*/+* (*n* = 11 mice) in two sequential cohorts including both males and females. Five *Plp1-creERT*/+; *Nf1*^*flox*^*/+* control mice were also injected for comparison. In addition, there were uninjected control mice housed in tamoxifen-free cages: *Plp1-creERT*/+; *R26-fs-GNAQ*^*Q209L*^/+ (*n* = 5), *Plp1-creERT*/+; *R26-fs-GNAQ*^Q209L^/+; *Nf1*^*flox*^*/+* (*n* = 2), and 35 of their cagemates of various other genotypes. The longest surviving mouse among those expressing *GNAQ*^*Q209L*^ was 72 weeks old, and therefore we aimed to age all other mice to at least 72 weeks. Nine of the control mice (of the 47 total) had to be euthanized between 50 to 63 weeks because of various problems (weight loss mostly but also head tilt, eye infection, or skin infection) but not tumors.

**Figure 1:**
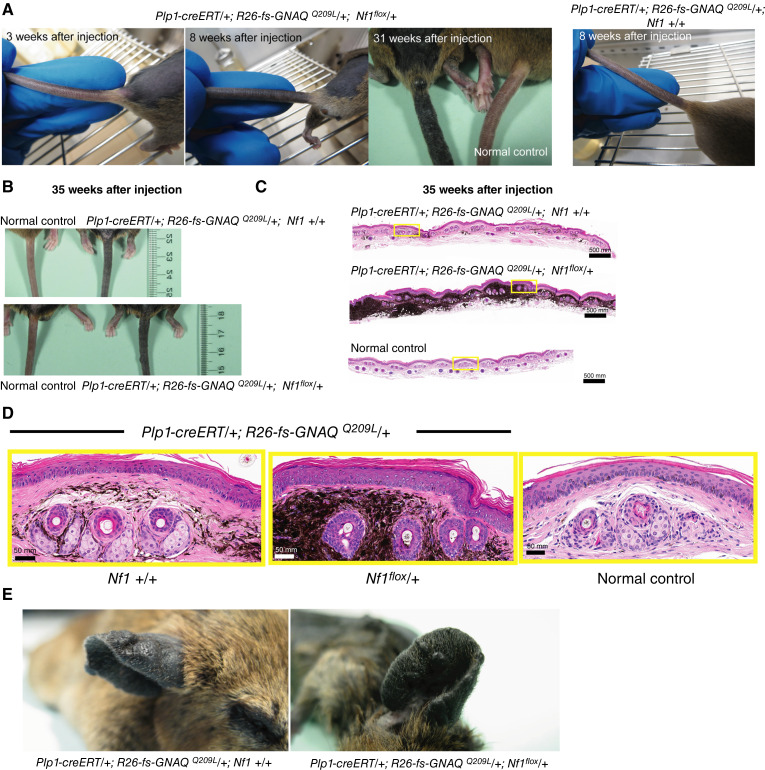
Effects of heterozygous *Nf1* loss on skin pigmentation in mice expressing oncogenic GNAQ. **A,** Tails of mice injected with tamoxifen at 5 weeks of age at various indicated time points after injection. The *Plp1-creERT/+*; *R26-fs-GNAQ*^*Q209L*^*/+*; *Nf1*^*flox*^*/+* mice developed skin hyperpigmentation 8 weeks after injection when the *Plp1-creERT/+*; *R26-fs-GNAQ*^*Q209L*^*/+*; *+/+* mice still looked normal. **B,** Hyperpigmented tail skin in both *Plp1-creERT/+*; *R26-fs-GNAQ*^Q209L^*/+*; *Nf1*^*flox*^*/+* and *Plp1-creERT/+*; *R26-fs-GNAQ*^*Q209L*^*/+*; *+/+* mouse tails at 35 weeks after injection, compared with that in their uninjected littermates. **C** and **D,** H&E analysis of tail skin from *Plp1-creERT/+*; *R26-fs-GNAQ*^*Q209L*^*/+*; *Nf1*^*flox/*^*+* and *Plp1-creERT/+*; *R26-fs-GNAQ*^*Q209L*^*/+*; *+/+* mice at 35 weeks after injection, with an uninjected control mouse tail skin section. Yellow boxes in **C** are enlarged in **D**. **E,** Hyperpigmented and thickened ear skin with small pigmented lesions in mice of the indicated genotypes.

The first noticeable phenotype in the *GNAQ*^*Q209L*^-expressing mice was hyperpigmented tail skin. This developed by 8 weeks after injection in the *Plp1-creERT*/+; *R26-fs-GNAQ*^*Q209L*^/+; *Nf1*^*flox*^*/+* mice and later in the *Plp1-creERT*/+; *R26-fs-GNAQ*^*Q209L*^/+; *+*/*+* mice (Fig. 1A). At 35 weeks after injection, *Plp1-creERT*/+; *R26-fs-GNAQ*^*Q209L*^/+; *Nf1*^*flox*^*/+* tail skin was much darker than *Plp1-creERT*/+; *R26-fs-GNAQ*^*Q209L*^/+; +/+ tail skin, which was darker than control tail skin (Fig. 1B–D). The excess pigmentation was located in the dermis. Most ears also developed small raised lesions (Fig. 1E), which grew slowly. One or more large tumors were found in 56% of the mice expressing *GNAQ*^*Q209L*^. Large tumors almost always appeared on the trunk and are defined in our study as those reaching at least 0.5 cm in diameter. We observed two types of large tumors, intradermal melanomas and a second type that we named “sparse pigment tumors.”

**Figure 2:**
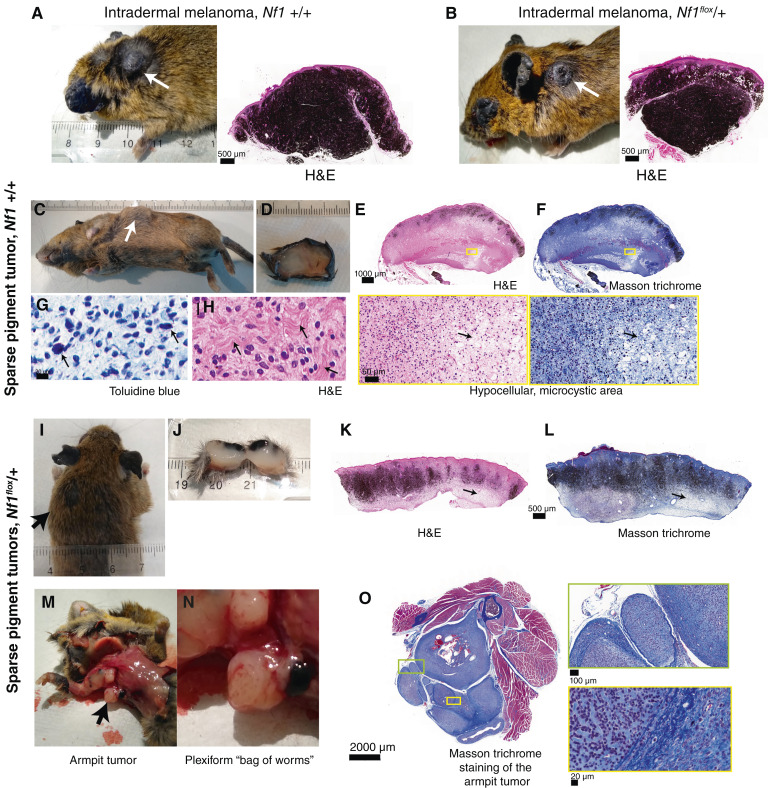
Histology of intradermal melanomas and sparse pigment tumors in mice expressing oncogenic GNAQ. **A,** 66 weeks after tamoxifen *Plp1-creERT/+*; *R26-fs-GNAQ*^*Q209L*^*/+*; *+/+* mouse (left) with an intradermal melanoma on the neck. The tumor is shown stained with H&E (right). **B,** 51 weeks after tamoxifen *Plp1-creERT/+*; *R26-fs-GNAQ*^*Q209L*^*/+*; *Nf1*^*flox*^*/+* mouse (left) with an intradermal melanoma on the upper back. The tumor is shown stained with H&E (right). **C–F,** 61 weeks after tamoxifen *Plp1-creERT/+*; *R26-fs-GNAQ*^*Q209L*^; *+/+* mouse (**C**) with a sparse pigment tumor on the side of the trunk; tumor shown just after removal (D) and sectioned and stained with H&E (E) and the Masson trichrome (**F**). An area containing both hypocellular and hypercellular regions of the tumor is enlarged in the boxes below. Arrows point to a hypocellular region. **G,** Close up images of granular mast cells (arrows) in toluidine blue–stained section of a sparse pigment tumor. **H,** Wavy collagen bundles (arrows) in H&E-stained section of sparse pigment tumor. **I,** 48 weeks after tamoxifen *Plp1-creERT/+*; *R26-fs-GNAQ*^*Q209L*^; *Nf1*^*flox*^*/+* mouse that developed a sparse pigment tumor on the left shoulder (arrow). **J,** Another sparse pigment tumor from a different *Plp1-creERT/+*; *R26-fs-GNAQ*^*Q209L*^; *Nf1*^*flox*^*/+* mouse, cut in half. **K** and **L,** The tumor in **I** was sectioned and stained with H&E (**K**) and the Masson trichrome (**L**). Arrows point to hypocellular region. **M** and **N,** The mouse in **I** also had an armpit tumor (arrow). This tumor had nodules with a macroscopic “bag of worms” plexiform appearance (**N**). **O,** The Masson trichrome staining of the nodule shown in **N**, with enlargements of areas of interest. The surrounding muscle and bone are included.

The intradermal melanomas were very similar to those we observed previously when we expressed *GNAQ*^*Q209L*^ in melanocytes and aged the mice (43, 45). The intradermal melanomas lost their overlying fur as they grew and were very darkly pigmented throughout (examples in Fig. 2A and B, with all tumors found shown in Supplementary Figs. S1A, S1B, S2A, and S2B). They were S100b-positive, which is typical for melanoma (Supplementary Fig. S3A and S3C). They also had many CD68-positive macrophages/melanophages (Supplementary Fig. S3B and S3D). There was no significant difference in the abundance of tumor cells or macrophages in *Nf1*^*flox*^*/+* tumors versus *Nf1* +/+ melanomas (Supplementary Fig. S3E and S3F).

The sparse pigment tumors were not similar to tumors seen before in mice, to the best of our knowledge. These tumors were intradermal, broad and flat, and did not disrupt the overlying fur. Macroscopic photographs of all sparse tumors that were found are presented in Supplementary Fig. S4A and S4B. Figure 2C–H shows the histology of one of these tumors in a *Plp1-creERT*/+; *R26-fs-GNAQ*^*Q209L*^/+; *+/+* mouse. When dissected from the body (Fig. 2D), the tumor was pigmented only on the upper surface and was otherwise gray colored. It also glistened and had a more rubbery texture than an intradermal melanoma. Alternating areas of increased and decreased cellularity were apparent in H&E-stained (Fig. 2E) and Masson trichrome (Fig. 2F)–stained sections. Additional H&E-stained tumor sections are shown in Supplementary Fig. S5A and S5B. Toluidine blue staining revealed granular mast cells (Fig. 2G). H&E staining showed bright pink and abundant wavy collagen filaments (Fig. 2H). Blood vessels were hyalinized (Supplementary Fig. S6A and S6B). *Plp1-creERT*/+; *R26-fs-GNAQ*^*Q209L*^/+; *Nf1*^*flox*^*/+* mice also exhibited sparse pigment tumors. Similar features were apparent at the macroscopic (Fig. 2I and J; Supplementary Fig. S4A and S4B) and microscopic levels (Fig. 2K and L; Supplementary Fig. S5A and S5B). The above features of the sparse pigment tumors are consistent with a Schwann cell–based tumor, such as a cutaneous neurofibroma. Histologic features associated with neurofibromas that we did not observe in sparse pigment tumors were whorls and verocay bodies (46).

In one interesting *Plp1-creERT*/+; *R26-fs-GNAQ*^*Q209L*^/+; *Nf1*^*flox*^*/+* mouse, two sparse pigment tumors developed in the dermis, one larger than the other (Fig. 2I). Around the same time, the mouse started holding up its right foreleg. It was euthanized, and necroscopy revealed a tumor in the right armpit as well (Fig. 2M). The armpit tumor was composed of three connected nodules, two of which had a plexiform “bag of worms” macroscopic appearance that is characteristic of plexiform neurofibromas (Fig. 2N). The Masson trichrome staining in Fig. 2O shows a possible connection of the nodule in Fig. 2N to the body, as well as some of the tumor lobes and structures making up the plexiform macroscopic appearance. Our working hypothesis is that these sparse pigment tumors arise from Schwann cells, and they are related to neurofibromas, schwannomas, or another kind of peripheral nerve sheath tumor. We return to the subject of the nature of sparse pigment tumors later in the “Results” section.

### Comparison of the intradermal tumor types

**Figure 3:**
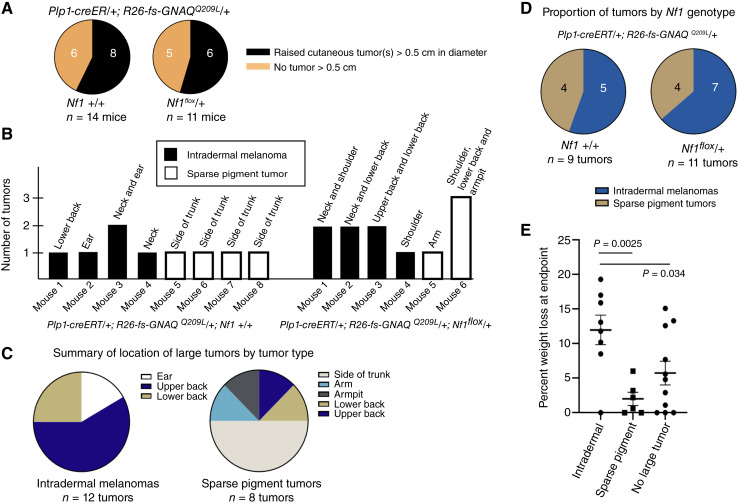
Comparison of cutaneous tumor numbers, location on the body, and effect on body weight. **A,** Proportion of mice with a large tumor by *Nf1* genotype. **B,** The number and location of large tumors in affected mice in study, organized by *Nf1* genotype. **C,** Summary of locations of large tumors by tumor type. **D,** Proportion of intradermal melanomas vs. sparse pigment tumors by *Nf1* genotype. **E,** Percent weight loss at mouse endpoint by tumor type or lack thereof.

As the mice were aging, we monitored them for body weight, general condition, and the presence of tumors. Of the 25 mice expressing *GNAQ*^*Q209L*^, we found that 14 (56%) developed one or more large tumors (>0.5 cm in diameter; 8/14 *Nf1* +/+ mice and 6/11 *Nf1*^*flox*^*/+* mice; Fig. 3A). Because almost all of the large tumors developed under the fur on the trunk, there were some that were not appreciated until their necroscopy was performed. We euthanized any mouse found with a large tumor soon after it was discovered, which ranged from 188 to 465 days after tamoxifen injection (Supplementary Table S1A, which also has additional details about mice in the study and identifies mice by ID numbers that match other figures in the supplementary material). The remaining mice in the study were euthanized for other reasons without finding a large tumor upon necroscopy. These reasons were piloerection, hunching, excessive scratching, and/or having severely affected ears.

The number of large tumors per affected mouse ranged from 1 to 3 (Fig. 3B). Interestingly, no mouse developed both intradermal melanoma and a sparse pigment tumor, but the cohort was not large enough to determine whether this was significant. We noted a trend in the locations of intradermal melanomas versus sparse pigment tumors. Intradermal melanomas were more frequently found around the head, neck, and upper back. This is similar to what has been observed in human cases of malignant blue nevi. In one published study of 10 lesions, the scalp was affected in five, the neck in one, and the trunk in four lesions (47). Mouse sparse pigment tumors were most frequently found on the sides of the trunk (Fig. 3C). The ratio of intradermal melanomas to sparse pigment tumors was similar in *Nf1* +/+ and *Nf1*^*flox*^/+ genotypes (Fig. 3D), and both types were found in males and females (Supplementary Table S1A).

Some of the mice exhibited significant weight loss. When examining the percent weight loss at endpoint, we found that the mice with intradermal melanomas lost more weight than the mice with sparse pigment tumors (*P* = 0.0025, unpaired *t* test), suggesting a more aggressive behavior of intradermal melanomas (Fig. 3E; Supplementary Table S1A). This is also consistent with the benign nature of most human cutaneous neurofibromas.

### *Nf1* loss accelerated intradermal melanoma tumorigenesis

**Figure 4:**
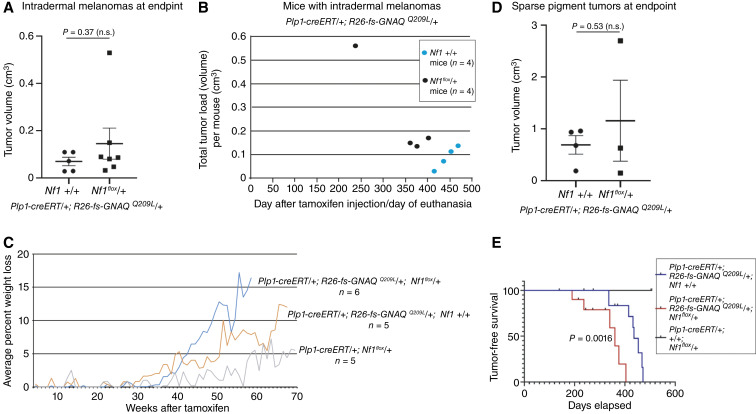
Intradermal melanoma tumorigenesis was accelerated by *Nf1* loss in mice expressing oncogenic GNAQ. **A,** Graph of intradermal tumor volumes at endpoint. Each point on the graph represents a different tumor. There was no significant difference between the average tumor volume in *Plp1-creERT*/+; *R26-fs-GNAQ*^*Q209L*^/+; *Nf1*^*flox*^/+ and *Plp1-creERT*/+; *R26-fs-GNAQ*^*Q209L*^/+; +/+ mice because mice were euthanized shortly after tumor discovery. Error bars represent the SEM. **B,** Graph of intradermal tumor volumes at endpoint, showing the combined tumor load per mouse on the *y* axis. Each dot represents a different mouse. The *x* axis indicates when each mouse was euthanized. **C,** Percent weight loss in the second cohort of mice over time in mice of the indicated genotypes. **D,** Graph of sparse pigment tumor volumes at endpoint. Each point on the graph represents a different tumor. There was no significant difference between the average tumor volumes in *Plp1-creERT*/+; *R26-fs-GNAQ*^*Q209L*^/+; *Nf1*^*flox*^/+ and *Plp1-creERT*/+; *R26-fs-GNAQ*^*Q209L*^/+; +/+ mice because mice were euthanized shortly after tumor discovery. Error bars represent the SEM. **E,** Kaplan–Meier plot of tumor-free survival in *Plp1-creERT*/+; *R26-fs-GNAQ*^*Q209L*^/+; *Nf1*^*flox*^/+ mice vs. *Plp1-creERT*/+; *R26-fs-GNAQ*^*Q209L*^/+; +/+ mice. There was a significantly decreased tumor-free survival in *Plp1-creERT*/+; *R26-fs-GNAQ*^*Q209L*^/+; *Nf1*^*flox*^/+ mice. n.s., not significant.

One of the primary research objectives of this study was to determine whether *Nf1* loss would accelerate tumorigenesis of intradermal melanoma in mice expressing *GNAQ*^*Q209L*^. This could be due to increased tumor initiation, an increased growth rate, or both. Because the tumors appeared over a fairly long time scale in the mice, started out under the fur, and grew rapidly once becoming noticeable, we do not have extensive measurements of tumor growth over time. However, we calculated the tumor volume at endpoint. Assuming no other confounding health factors, the tumors would all have been harvested at a similar size by following our ethics protocol. This was essentially what was done, because there was no significant difference in the average individual tumor volume between the *Plp1-creERT/+*; *R26-fs-GNAQ*^*Q209L*^*/+*; *Nf1* +/+ and *Nf1*^*flox*^/+ intradermal melanomas at euthanasia (Fig. 4A; Supplementary Table S1B). However, all *Nf1*^*flox*^/+ mice with intradermal melanoma were euthanized by day 404 of the study before the first *Nf1* +/+ mouse with intradermal melanoma was euthanized on day 416 (Fig. 4B; Supplementary Table S1A). In addition, if the total intradermal melanoma burden per mouse in volume is summed up, all *Nf1*^*flox*^/+ mice exhibited equivalent or greater volumes than all *Nf1* +/+ mice, because most of the *Nf1*^*flox*^/+ mice had more than one simultaneous intradermal melanoma (Fig. 3B). *Nf1*^*flox*^*/+* mice also lost weight more quickly than *Nf1+/+* mice, which is consistent with accelerated intradermal melanoma development (Fig. 4C).

Similarly, there was no significant difference between the average tumor volume of the sparse pigment tumors in *Nf1* +/+ and *Nf1*^*flox*^/+ mice at euthanasia (Fig. 4D; Supplementary Table S1B). Kaplan–Meier analysis including all *GNAQ*^Q209L^-expressing mice in the study showed that *Nf1*^*flox*^*/+* significantly decreased tumor-free survival (including both dermal tumor types together, *P* = 0.0016; Fig. 4E). As mentioned above, mice were euthanized shortly after tumor discovery. Hence, all available evidence points to *Nf1* haploinsufficiency stimulating intradermal melanoma tumorigenesis.

### *Nf1* loss increased the size of uveal melanoma in the mouse eye

**Figure 5:**
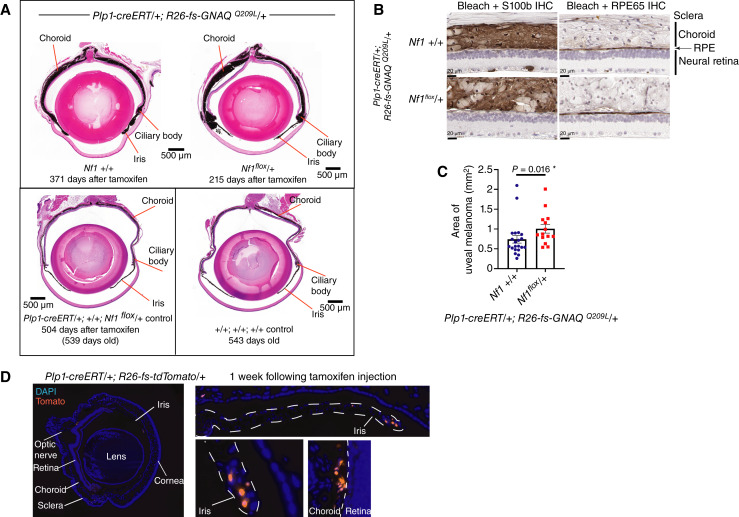
*Nf1* loss increased the size of uveal melanoma tumors in mice expressing oncogenic GNAQ. **A,** H&E-stained sections of representative eyes of the indicated genotypes. **B,** IHC to determine whether the excess pigmented tissue in eyes was uveal melanoma, using IHC to examine expression of S100B (for melanocytes, left) or RPE65 (for retinal pigment epithelium “RPE,” right). The excess tissue was S100B-positive, and the RPE was a normal, single-layered epithelium. **C,** Quantification of the area of uveal melanoma in *Plp1-creERT*/+; *R26-fs-GNAQ*^*Q209L*^/+; *Nf1*^*flox*^/+ vs. *Plp1-creERT*/+; *R26-fs-GNAQ*^*Q209L*^/+; *Nf1* +/+ eyes. All eyes examined were affected. There was a significant increase in area in the *Nf1*^*flox*^*/+* eyes. **D,** 6-week *Plp1-creERT/+*; *Rosa26-fs-tdTomato/+* eye one week following injection with tamoxifen. *Plp1-creERT* induced tomato expression (red) in cells in the uveal tract, including the iris, ciliary body, and choroid (shown in enlargements, right). Sections were counterstained with 4',6-diamidino-2-phenylindole (DAPI; blue).

We assessed the eyes in the mice as they were euthanized, as described above. We sectioned and stained sections taken from the middle of the eyes with H&E. In 100% of mice expressing *GNAQ*^*Q209L*^, there was an abnormal expansion of the area of pigmented tissue, which includes the choroid, ciliary body, and iris, otherwise known as the uveal tract (Fig. 5A, top row). The uveal tracts of the five tamoxifen-injected *Plp-creER/+*; *Nf1*^*flox*^*/+* control mice were normal (example in Fig. 5A, bottom row). We bleached sections to remove melanin and performed IHC for S100b (for melanocytes) or RPE65 (for the retinal pigment epithelium) to confirm that the excess pigmented tissue was from the uveal tract (Fig. 5B). An antibody specific to RPE65 stained an expected narrow strip of cells between the neural retina and the choroid. The expanded choroid, ciliary body, and iris were positive for S100b and negative for RPE65, consistent with the development of uveal melanoma.

The size of the uveal melanoma in each eye section was quantified using ImageJ. There was a significant increase in the average area in the *Nf1*^*flox*^/+ eyes compared with that in the *Nf1* +/+ eyes (*P* = 0.016, Fig. 5C). This is despite the fact that the tumor tissue in the *Nf1* +/+ mice had a longer time to grow, on average. Therefore, we conclude that *Nf1* loss promotes uveal melanoma driven by oncogenic GNAQ.

To identify the *Plp1-creERT*–expressing cells in the eyes, we crossed *Plp1-creERT* to the *R26-fs-tdTomato* reporter line [*Gt(ROSA)26Sor*^*tm14(CAG-tdTomato)Hze*^] to label expressing cells with tdTomato. We administered tamoxifen by the same intraperitoneal injection schedule at 5 weeks of age and collected the eyes at 6 weeks. There were tdTomato-positive cells in the iris and choroid of the eyes but not in the sclera or retina, in which melanocytes are not found (Fig. 5D). Hence, *Plp1-creERT* can be used to induce uveal melanoma from melanocytes in the uveal tract when tamoxifen is given at 5 weeks of age.

### Other pigmented lesions in the mice

Oncogenic GNAQ also causes a variety of other pigment cell defects. *GNAQ* is frequently mutant in primary melanoma of the CNS (refs. 8, 43). To assess the pigmented lesions associated with the CNS in the mice, we photographed the spines and collected the brains during necroscopy of the second cohort of the *Plp1-creERT/+*; *R26-fs-GNAQ*^*Q209L*^/+ mice, with and without conditional *Nf1/+* loss. All mice expressing *GNAQ*^*Q209L*^exhibited small pigmented lesions in the lumbar area of the back centered above the spine (Supplementary Fig. S7A and S7B). There were also multiple examples of mice with pigmented lesions within the spine upon sectioning (Supplementary Fig. S7C). However, there was no significant difference between *Nf1*^*flox*^*/+* and +/+ mice. In addition, we noted lesions on the ventral brain surface, but again there was no significant difference between *Nf1*^*flox*^*/+* and +/+ mice with respect to lesion size (Supplementary Fig. S7D and S7E). Compared with the *Mitf-cre*/+; *R26-fs-GNAQ*^*Q209L*^/+ model described previously (26, 45), the CNS lesions were much smaller using *Plp1-creERT*, and there was also no hyperactivity or head tossing.

*GNAQ* ^*Q209L*^-expressing mice also develop pigmented lung lesions. It is difficult to ascertain whether these are distant metastases, but there are no known resident melanocytes in the lungs (26). In animals expressing *GNAQ*^*Q209L*^, there were on average 19 lung lesions per mouse, regardless of the *Nf1* genotype (Supplementary Fig. S8A). There was no difference in the average size of the lung lesions in the *Nf1* +/+ versus *Nf1*^*flox*^*/+* mice. These lesions were all small, less than 0.13 mm^2^ in area (Supplementary Fig. S8B–S8D).

Lastly, we examined the phenotype of uninjected mice of the mutant genotypes. We previously reported that there is some limited activation of CreERT in *Plp1-creERT/+*; *R26-fs-GNAQ*^*Q209L*^/+ mice in the absence of any tamoxifen (*i.e.*, “leaky CreERT activity”; ref. 43). This small level of activity can be detected because changes in pigmentation by *GNAQ*^*Q209L*^ are easy to spot. In this study, we examined two *Plp1-creERT*/+; *R26-fs-GNAQ*^*Q209L*^/+; *Nf1*^*flox*^*/+* mice and five *Plp1-creERT*/+; *R26-fs-GNAQ*^*Q209L*^/+; *+/+* mice, housed in tamoxifen-free cages, after aging to 72 weeks. As in ref. 43, there were tiny punctate spots on all shaved trunks. In addition, two tails exhibited small pigmented lesions in the dermis (Supplementary Fig. S9B and S9C, control shown in Supplementary Fig. S9A), one eye had an expanded pigmented layer (Supplementary Fig. S10B, controls shown in Supplementary Fig. S10A, S10C, and S10D), and one spine had an overlying lesion within the muscle (whole-mount image in Supplementary Fig. S11A and H&E-stained section in Supplementary Fig. S11B).

### Effects of *Nf1* loss on the melanoma transcriptome

We next performed bulk RNA-seq to identify DE genes caused by *Nf1* haploinsufficiency. We used all *GNAQ*^*Q209L*^-expressing mice in the second cohort of breeding for this purpose (Supplementary Table S1A). Dermal tumor tissue was collected for RNA extraction when each mouse was euthanized for a dermal tumor, as previously described. The dermal tumors seemed to be at a consistent stage upon collection, as judged by their macroscopic appearance (Supplementary Figs. S1A, S1B, S4A, and S4B) and an H&E-stained section of a separate piece of each tumor (Supplementary Figs. S2A, S2B, S5A, and S5B). At the same time, one eye was removed from each mouse, and fine forceps were used to dissect out uveal melanoma tissue for RNA extraction. The other eye was embedded for histology.

**Figure 6:**
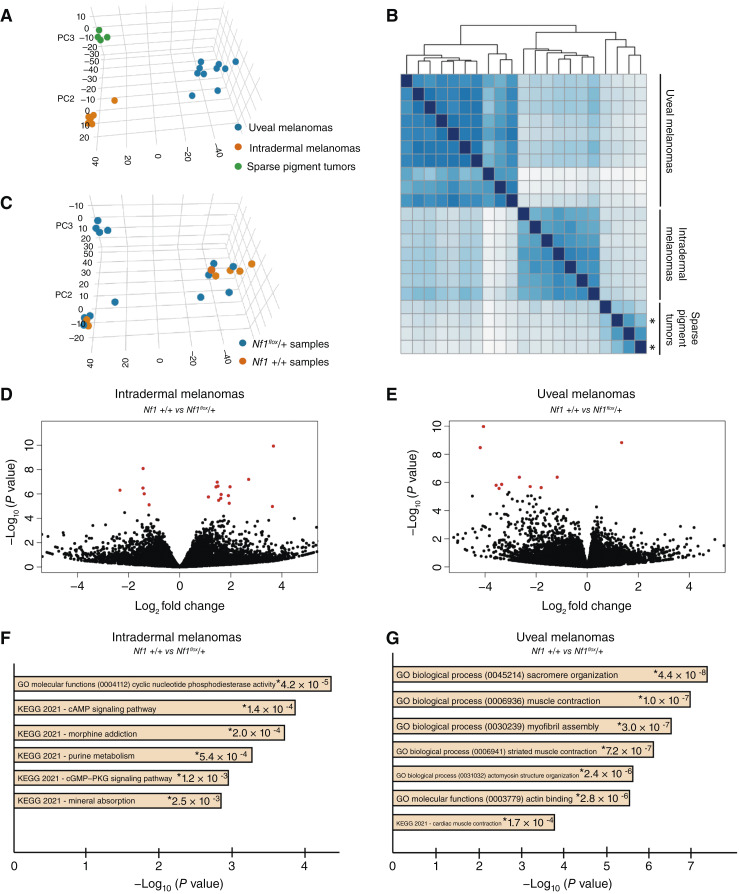
Transcriptomic analysis of uveal melanoma, intradermal melanoma, and sparse pigment tumors. **A** and **B,** All samples were included in unsupervised clustering by gene expression, regardless of the *Nf1* genotype or tumor type. Principal component analysis (**A**) and the sample distance dendrogram (**B**) show that the three different tumor types cluster separately from each other. In **B**, the two sparse pigment tumors (shown in Fig. 2I and M) from the same mouse are indicated with asterisks. **C,** Same principal component analysis plot as shown in **A** but now color coded to show the *Nf1* genotypes. There was no obvious clustering by *Nf1* genotype within tumor types. **D** and **E,** Volcano plots of differential gene expression in *Nf1*^*flox*^/+ vs. *Nf1 +/+* intradermal melanomas (**D**) and uveal melanomas (**E**). **F** and **G,** Top terms found in GO analysis of the DE genes in intradermal melanoma (**F**) and uveal melanoma (**G**). KEGG, Kyoto Encyclopedia of Genes and Genomes.

We included 7 intradermal melanomas (3 *Nf1* +/+ and 4 *Nf1*^*flox*^/+), 4 sparse pigment tumors (all *Nf1*^*flox*^/+), and 10 uveal melanomas (5 *Nf1* +/+ and 5 *Nf1*^*flox*^/+). RNA-seq was performed on all 21 RNA samples in one run. We used DESeq2 to compare the samples in several different analyses, as described below. To assess overall relationships, we included all samples for unsupervised clustering by gene expression, regardless of the *Nf1* genotype or tumor type. In the principal component analysis (Fig. 6A) and sample distance dendrogram (Fig. 6B), the three tumor types clustered separately. We included the plexiform armpit tumor and a dermal sparse pigment tumor from the same mouse for RNA-seq (indicated by asterisks in Fig. 6B). This was the mouse shown in Fig. 2I and M. The armpit tumor grouped with the other sparse pigment tumors in gene expression despite its different location in the body. The *Nf1* genotypes are indicated by color in the same principal component analysis plot in Fig. 6C, which revealed no strong pattern of clustering by genotype compared with the significant differences produced by tumor type.

We then performed DE analysis to compare *Nf1*^*flox*^/+ versus *Nf1 +/+* tumors within tumor types. Volcano plots of the results are shown in Fig. 6D (intradermal melanoma) and Fig. 6E (uveal melanomas). In the intradermal melanomas, there were 15 upregulated genes and 10 downregulated genes in the *Nf1*^*flox*^*/*+ tumors compared with *Nf1* +/+, with *P*_adj_ <0.05 (Supplementary Table S1C). Among these genes, the most significant term was GO molecular function: 0004112, “cyclic nucleotide phosphodiesterase (PDE) activity,” *P* = 4.2 × 10^−5^ (Fig. 6F; Supplementary Table S1D). This was supported by the upregulation of *Adcy1* and *Atp1b2* and the downregulation of *Pde10a* and *Pde3a* in the *Nf1*^*flox*^/+ tumors. cAMP levels are regulated by adenylyl cyclases, which produce cAMP from ATP, and by the activity of PDEs, which hydrolyze and degrade cyclic nucleotides (cAMP and cGMP). The balance between the activity of these two enzymatic families controls the activation of the downstream effectors, such as protein kinase A and the cAMP-responsive element–binding protein.

In the uveal melanomas, there were 23 downregulated genes and 2 upregulated DE genes with *P*_adj_ < 0.05 (Supplementary Table S1E). There was an exceptionally clear signal from the *Nf1*-mutant uveal melanomas in GO analysis (Fig. 6G; Supplementary Table S1F). Of the 23 downregulated genes, 20 were related to muscle contraction and/or myogenesis. The most significant term was GO biological process: 0045214, “sarcomere organization,” *P* = 4.4 × 10^−8^. The complete list of DE genes relating to muscle function is as follows: *Acta1*, *Actn3*, *Atp2a1*, *Casq1*, *Ckm*, *Cmya5*, *Cox6a2*, *Dhrs7c*, *Mybpc2*, *Myh4*, *Mylk2*, *Myom2*, *Myoz1*, *Pgam2*, *Phkg1*, *Pvalb*, *Synpo2*, *Tnni2*, *Tnnt3*, and *Tpm2*. A discussion of the relevance of cAMP and muscle gene expression changes can be found in the “Discussion” section. There was only one DE gene that was found in both melanoma tumor types when *Nf1* was mutant. This was *Rpl26*, which encodes a large subunit ribosomal protein. Although RPL26 has been previously linked to cancer through the regulation of p53, the prediction is that *Rpl26* downregulation would promote tumorigenesis, and *Rpl26* was upregulated in our datasets (48).

Although uveal melanoma prognosis is very strongly correlated with monosomy 3 and the loss of *BAP1*, we wondered whether any of the DE genes identified above were correlated with survival in the TCGA-UVM database. In fact, 20% of these DE genes (9/49) were significantly correlated with uveal melanoma survival, suggesting there could be some overlap in prognosis-related targets by different genetic pathways (Supplementary Fig. S12A–S12D). For all but one correlated gene, the relationship was in the expected direction (e.g., if the gene was upregulated in the *Nf1*^*flox*^*/+* mouse tumors, then higher expression was correlated with a worse outcome in human patients). Two highly significant correlations were observed for the muscle *COX6A2* gene (*P* = 1.7 × 10^−5^) and for *ADCY1* (*P* = 7.5 × 10^−5^). Also, a very significant correlation was found for the upregulated *GFRA2* gene (*P* = 5.9 × 10^−6^). The expression of *GRFA2* designates a neural crest stem cell signature found in cutaneous melanomas (49).

### The sparse pigment tumors are likely neurofibromas

**Figure 7:**
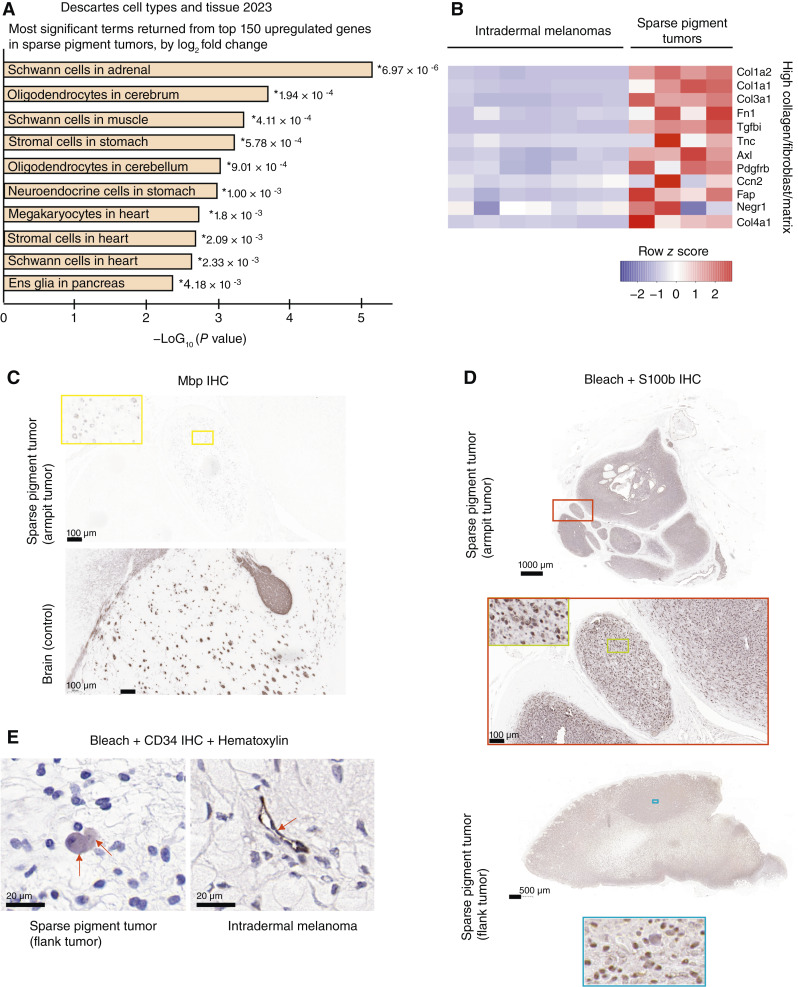
Gene expression analysis suggests that sparse pigment tumors are related to Schwann cells. **A,** The most significant terms returned from GO analysis of the top 150 genes upregulated in sparse pigment tumors vs. intradermal melanomas, by log_2_ fold change, using Descartes cell types and tissues, 2023. **B,** Heatmap of gene expression that indicates higher collagen, fibroblast, and matrix gene expression in sparse pigment tumors. **C–E,** IHC for Mbp, a marker for Schwann cells (**C**), or S100b, a marker for neural crest–derived cells (**D**), or CD34 (**E**) in indicated tissues. Red arrows in **E** indicate cells of interest. The CD34 IHC gave different results in the two tumor types. CD34-positive cells in the intradermal melanoma line capillaries and vessels. In the sparse pigment tumors, arrows indicate examples of weak CD34-positive cells that are also round and basophilic. Ens, enteric nervous system.

We then compared gene expression in the intradermal melanomas and sparse pigment tumors, irrespective of *Nf1* genotype, in order to better define their differences. There were 7,350 DE genes with a *P*_adj_ cut off <0.05 (Supplementary Table S1G). We first considered the top genes that were most differentially expressed by log_2_ fold change. Many well-known pigmentation genes were among the 150 most upregulated genes in the intradermal melanomas: *Slc45a2*, *Tyrp1*, *Tyr*, *Pmel*, *Dct*, *Oca2*, *Mlana*, *Slc24a4*, and *Mc1r*. On the other hand, many Schwann cell/oligodendrocyte/glia-supporting genes were in the top 150 genes upregulated in the sparse pigment tumors: *Gpr17*, *Crispld1*, *Ptprz1*, *Col20a1*, *Scn7a*, *Lrrn1*, *Wnt16*, *Matn4*, *Asic4*, *Mog*, *Nkx2-2*, *Kirrel3*, *Tenm3*, *Kcnh8*, *Dbh*, *Srcin1*, and *Plxnb3* (Supplementary Table S1H). We also looked up various genes classically expressed by Schwann cell precursors or Schwann cells and found that *Gap43*, *Fabp7*, *Mpz*, *Dhh*, *Ngfr*, *Ncam1*, and *Mbp* were all significantly upregulated in the sparse pigment tumors than in the intradermal melanomas, as was *Plp1*. The top 150 upregulated genes in the sparse pigment tumors returned the significantly enriched terms, “Schwann cells in adrenal,” “oligodendrocytes in cerebrum,” “Schwann cells in muscle,” “oligodendrocytes in cerebellum,” *etc*. (Fig. 7A). This supports the hypothesis that the sparse pigment tumors are some kind of nerve sheath neoplasm.

Miskolczi and colleagues (50) found that the presence of fibroblast tumor growth factor-β suppressed YAP/PAX3-mediated MITF expression and was associated with a dedifferentiated phenotype in cutaneous melanoma. We noticed that the second most significant DE gene in our comparison was *Tgfbi* (TGF-β induced), which had a log_2_ fold change of 4.9 (upregulated in sparse pigment tumors) and a *P*_adj_ value of 7.5 × 10^−86^. *Mitf*, on the other hand, was downregulated in the sparse pigment tumors with a log_2_ fold change of −3.15 and a *P*_adj_ value of 1.23 × 10^−15^. Other genes in the suggested collagen high/fibroblast/matrix gene signature proposed in ref. 50 were upregulated in the sparse pigment tumors compared with the intradermal melanomas (genes in heatmap, Fig. 7B). This could reflect a greater contribution of fibroblasts and the extracellular matrix in the sparse pigment tumors.

Mbp is a marker for peripheral glial cells. The only sparse pigment tumor with Mbp positivity was the armpit tumor (Fig. 7C). Some of the cells in this tumor expressed Mbp in a ring, such as might be expected in a myelinated nerve. Nerve bundles can become entrapped in plexiform neurofibromas. Mbp expression was weaker than that in a positive control normal mouse brain (Fig. 7C). In contrast, there was widespread and clear S100b staining in all of the sparse pigment tumors (examples shown in Fig. 7D). S100b expression has been noted as a consistent marker for peripheral nerve sheath tumors. We were also interested in CD34, which has been proposed to distinguish neurofibroma from desmoplastic melanoma by a fingerprint pattern of expression in the neurofibromas (46). In the mouse intradermal melanomas, CD34-positive staining was found in cells lining blood vessels, as mentioned in ref. 46 (Fig. 7E). An endothelial pattern was not clear in the sparse pigment tumors. Instead, there may have been weak CD34 expression in round basophillic cells of unknown identity, which were scattered throughout (Fig. 7E). In this way, CD34 was differential between the two tumor types but not in a previously reported pattern.

Given the strong similarity between neurofibromas and mouse sparse pigment tumors, combined with the fact that *GNAQ*^Q209L^ alone was able to stimulate their formation, we investigated the cBioPortal for Cancer Genomics database to search for mutations in *GNAQ* or *GNA11*. In the nerve sheath tumors (Johns Hopkins, 2024) dataset (51), 2 of 54 plexiform neurofibromas had a *GNAQ*^*T96S*^mutation, a known oncogenic hotspot (52) that induces a gain of activity (https://www.cbioportal.org/study/summary?id=nst_nfosi_ntap). The mutation was present in one plexiform neurofibroma each from two different patients. This finding, along with previously mentioned data, strongly suggests that mouse sparse pigment tumors are closely related to neurofibromas.

## Discussion

MAPK pathway activation is one of the key events in melanoma, as well as in many other cancers (1). Mutations in MAPK pathway components in sun-exposed melanoma occur in the oncogenes, *BRAF* and *NRAS*, and the tumor suppressors *RASA2* and *NF1* (2–5). The net intensity of MAPK signaling in a cell is determined by the cumulative activity of components all along the pathway. In sun-exposed skin, melanocytic nevi begin with a single activating MAPK mutation, most often in *BRAF*. Multiple and independent genetic alterations can incrementally accumulate over time, building to higher levels of MAPK signaling in melanoma (13, 53).

In melanomas that arise outside of an epithelium, such as in the dermis and eye, MAPK pathway activation is most frequently achieved through the activation of Gα_q/11_ signaling, rather than directly through *BRAF* or *NRAS*. Gα_q/11_ activates phospholipase C β, stimulating PKC and feeding into the MAPK pathway through RASGRP3 (10). A tumor-promoting effect of neurofibromin loss in nonepithelial melanoma has been suggested by a greater than expected frequency of uveal melanoma in patients with neurofibromatosis type 1 (heterozygous germline *NF1* mutation carriers; ref. 20). We also previously described a patient with neurofibromatosis type 1 who developed a *GNAQ*^*Q209P*^-mutant uveal melanoma (18). It should also be noted that somatic mutations in *NF1* are frequent in a rare type of melanoma known as desmoplastic melanoma. This type of melanoma is primarily located in the dermis, but some cases have an overlying lentigo maligna component in the epidermis (54). Shain and colleagues (36, 55) reported that 54% of desmoplastic melanomas are *NF1* mutant. In about half of these cases, *NF1* was heterozygous. Desmoplastic melanoma is characterized by unpigmented, spindle-shaped melanocytes surrounded by an abundant fibrous collagen stroma. However, *GNAQ* or *GNA11* mutations have never been reported in this type of melanoma.

To address whether mutations in *NF1* might synergize with oncogenic Gα_q_ to promote nonepithelial melanomagenesis, we first surveyed the published literature and the TCGA-UVM dataset. We found that heterozygous partial loss of chromosome 17, including the *NF1* gene, recurs in uveal melanoma (2.5%) and blue nevus–type intradermal melanoma (14%). There were more details about the uveal melanoma cases, which shared some striking similarities (39). Both cases carried a *GNAQ*^*Q209P*^ mutation and seemed to be normal for *BAP1*, *EIF1AX*, and *SF3B1*. They were both placed in copy-number cluster 1, despite lacking CNAs on chromosome 6 (39). In addition, one of these cases carried a *RASA2* mutation (*Rasa2*^*K81Q*^), the only one in the dataset. This is significant because half of *RASA2*-mutant sun-exposed melanomas exhibit a co-occurring mutation in *NF1* (5, 12).

To directly test the interaction between neurofibromin and Gα_q/11_ in a model system, we studied the effects of conditional *Nf1* loss in mice expressing human oncogenic *GNAQ*^Q209L^ (26). Because the loss of *NF1* in the human melanoma cases was heterozygous, we generated *Nf1* haploinsufficiency in the mice. We chose to use the *Plp1-creERT* transgene [*Tg(Plp1-cre/ERT)3Pop*] for this. This tamoxifen-inducible CreERT line is expressed in peripheral glia (Schwann cells) and melanocytes and was of interest to us due to its previous connections with *Nf1* and tumorigenesis (22, 40–44). *Plp1-creERT* has not been used before in postnatal mice to drive *R26-fs-GNAQ*^*Q209L*^; however, we previously described CNS melanoma with tamoxifen injections early in embryogenesis (43). In this study, we injected tamoxifen at 5 weeks old.

As expected, the *GNAQ*^*Q209L*^-expressing mice developed intradermal melanomas (26, 45). The loss of one copy of *Nf1* accelerated the formation and/or growth of intradermal melanomas, as these tumors were detected earlier in the *Nf1*^*flox*^/+ mice. Although we could not track uveal melanoma in the eyes of living mice, all *GNAQ*^*Q209L*^-expressing mice exhibited uveal melanoma at euthanasia, with the average area of uveal melanoma significantly larger in *Nf1*^*flox*^*/+* eye sections, despite having had less time to develop, due to earlier cutaneous tumor formation or other health-related criteria necessitating euthanasia. Ideally, the role of *Nf1* in melanoma would be studied separately in mice with only uveal melanoma or only intradermal melanoma. This could be done in the future by using the *Mitf-Cre* line or perhaps by changing the timing of tamoxifen with *Plp1-creERT* (26, 43).

We identified DE genes that were produced by *Nf1* heterozygous loss in melanoma driven by *GNAQ*^*Q209L*^. In the intradermal melanomas, the most significant GO term was “Cyclic nucleotide PDE activity,” *P* = 4.2 × 10^−5^. This was supported by the upregulation of *Adcy1* and *Atp1b2* and the downregulation of *Pde10a* and *Pde3a* in the *Nf1*^*flox*^/+ tumors. cAMP levels are regulated by adenylyl cyclases, which produce cAMP from ATP, and by the activity of PDEs, which hydrolyze and degrade cyclic nucleotides (cAMP and cGMP). The balance between the activity of these two enzymatic families controls the activation of the downstream effectors, protein kinase A (PKA) and the cAMP-responsive element–binding protein. We found that high-*ADCY1* expression is very strongly associated with decreased survival in patients with uveal melanoma (*P* = 7.5 × 10^−5^). cAMP signaling has not been specifically investigated in nonepithelial melanomas, but otherwise has been the subject of much work in the melanocyte field (reviewed in ref. 56). In melanocytes, *NF1* inactivation was previously linked to increased activity of cAMP-mediated PKA and ERK, which led to the overexpression of Mitf (16).

In the mouse uveal melanomas, almost all of the DE genes in *Nf1*^*flox*^*/+* tumors were downregulated and many were related to muscle function. The downregulated *COX6A* muscle gene was very significantly associated with worse survival in patients with uveal melanoma (*P* = 1.7 × 10^−5^). *COX6A* encodes a subunit of the cytochrome c oxidase complex, the last enzyme in the mitochondrial electron transport chain. The downregulation of *COX6A* could therefore affect tumor metabolism. There are also some interesting muscle connections in the literature. For example, neurofibromin has been shown to be required for skeletal muscle development and function in mice (57), and individuals with neurofibromatosis type 1 experience hypotonia, decreased strength, and reduced motor function (58). Most intriguingly, people with myotonic dystrophy, which is caused by autosomal dominant repeat expansions disrupting the first exons of the muscle genes *DMPK* (in type 1) or *CNBP* (in type 2), develop a significantly elevated number of thyroid, endometrium, ovary, melanoma, colon/rectum, and testis cancers (59). One study found a 28-fold increased risk of uveal melanoma in people with myotonic dystrophy type 1 (60). *CNBP* is located on chromosome 3, which is frequently lost in uveal melanoma. We checked the association between *DMPK* and *CNBP* expression and survival in the TCGA-UVM dataset. Low *DMPK* expression had a nearly significant association with reduced survival (*P* = 0.054), and low *CNBP* expression also trended in that direction (*P* = 0.16).

In addition, a new cutaneous tumor type was produced in the mice, which we called sparse pigment tumors. Although also intradermal, these tumors were deeper, did not disrupt the overlying hair, and had features suggestive of Schwann cell–based neoplasms, such as a neurofibroma, schwannoma, or other peripheral nerve sheath tumors. These features were the macroscopic appearance, the combination of dermal and plexiform presentations, alternating areas of hyper- and hypo-cellularity, strong S100b positivity, the presence of mast cells, collagen bundles, and hyalinized vessels, and the upregulation of Schwann cell–specific gene expression compared with those in the intradermal melanomas. On the other hand, these tumors lacked a whorly organization, verocay bodies, and CD34-positive fingerprints, which are often found in neurofibromas. We then investigated the cBioPortal for Cancer Genomics database to search for mutations in *GNAQ* or *GNA11* in Schwann cell–based tumors. In the nerve sheath tumors (Johns Hopkins, 2024) dataset (51), 2 of 54 plexiform neurofibromas had a *GNAQ*^*T96S*^ mutation, a known oncogenic hotspot in hepatocellular carcinoma. *In silico* structural analysis done by Choi and colleagues (52) indicated that the T96S mutation may destabilize the interaction between the regulator of G protein signaling protein and GNAQ, reducing the inhibitory effect of the regulator of G protein signaling protein on GNAQ signaling and causing a gain of function. These T96S hotspot mutations in plexiform neurofibroma corroborate our findings in mice that oncogenic GNAQ can transform Schwann cells. Chen and colleagues (61) reported that the activation of Yap in Schwann cells through knockout of *Lats1* and *Lats2* drove neurofibroma formation in mice when combined with *Nf1* loss, which drives MAPK signaling. In melanocytes, we know that Gα_q/11_ activates both Yap and MAPK signaling, and this could be the reason that *GNAQ*^*Q209L*^ alone was sufficient to transform Schwann cells (6, 26, 62, 63). Our results here suggest that the *Plp1-creERT; GNAQ*^*Q209L*^ model with tamoxifen at 5 weeks may be useful as a preclinical model for neurofibroma.

Also of interest are malignant melanotic nerve sheath tumors (MMNST), a tumor type redefined in the 2021 World Health Organization classification of tumors of the CNS (previously called melanotic schwannoma). These are rare and aggressive neoplasms that frequently have loss-of-function mutations in the *PRKAR1A* gene, which encodes a negative regulator subunit of PKA. Terry and colleagues (64) recently published a case report of a woman with a MMNST that contained both a *PRKAR1A* frameshift mutation and a *GNAQ*^*R183L*^oncogenic hotspot mutation. Our results support their hypothesis that GNAQ activation can promote tumorigenesis in Schwann cells. Also, malignant peripheral nerve sheath tumor cell lines derived from individuals with neurofibromatosis type 1 were found to have basal cAMP levels twofold higher than those in normal Schwann cells (65). cAMP binds to PRKAR1A, which triggers conformational changes that dissociate PRKAR1A from the rest of the PKA complex, releasing its repression. Therefore, upregulating PKA activity might be a common tumor-promoting switch for both Schwann cells and melanocytes existing in dermal-like environments.

In summary, neurofibromin is a very large and complex tumor suppressor, the loss of which is known to contribute to the formation of melanoma in the skin and cause neurofibromas, Schwann cell–based tumors. Heterozygous 17q11.2 loss that includes the *NF1* locus is an uncommon, but recurrent phenomenon in intradermal and uveal melanomas that we think should be considered a potentially significant finding. In addition, our mouse model provides important evidence that oncogenic GNAQ in postnatal *Plp1*-expressing cells causes nerve sheath–like neoplasms, which should be further investigated as oncogenic hotspot mutations in *GNAQ* have now been found in plexiform neurofibromas and MMNSTs.

## Supplementary Material

Supplementary Figure 1 Supplementary Figure 1

Supplementary Figure 2 Supplementary Figure 2

Supplementary Figure 3 Supplementary Figure 3

Supplementary Figure 4 Supplementary Figure 4

Supplementary Figure 5 Supplementary Figure 5

Supplementary Figure 6 Supplementary Figure 6

Supplementary Figure 7 Supplementary Figure 7

Supplementary Figure 8 Supplementary Figure 8

Supplementary Figure 9 Supplementary Figure 9

Supplementary Figure 10 Supplementary Figure 10

Supplementary Figure 11 Supplementary Figure 11

Supplementary Figure 12 Supplementary Figure 12

Supplementary Table 1 Supplementary Table 1a-1h
