# Supplementary material for: Loss of NF1 Accelerates Uveal and Intradermal Melanoma Tumorigenesis, and Oncogenic GNAQ Transforms Schwann Cells
Source: Cancer Res Commun. 2025 Feb 3;5(2):209–25. doi: 10.1158/2767-9764.CRC-24-0386 (PMC11788999; doi:10.1158/2767-9764.CRC-24-0386)
Supplement: Supplementary Figure 2 [file crc-24-0386_supplementary_figure_2_suppsf2.pdf]

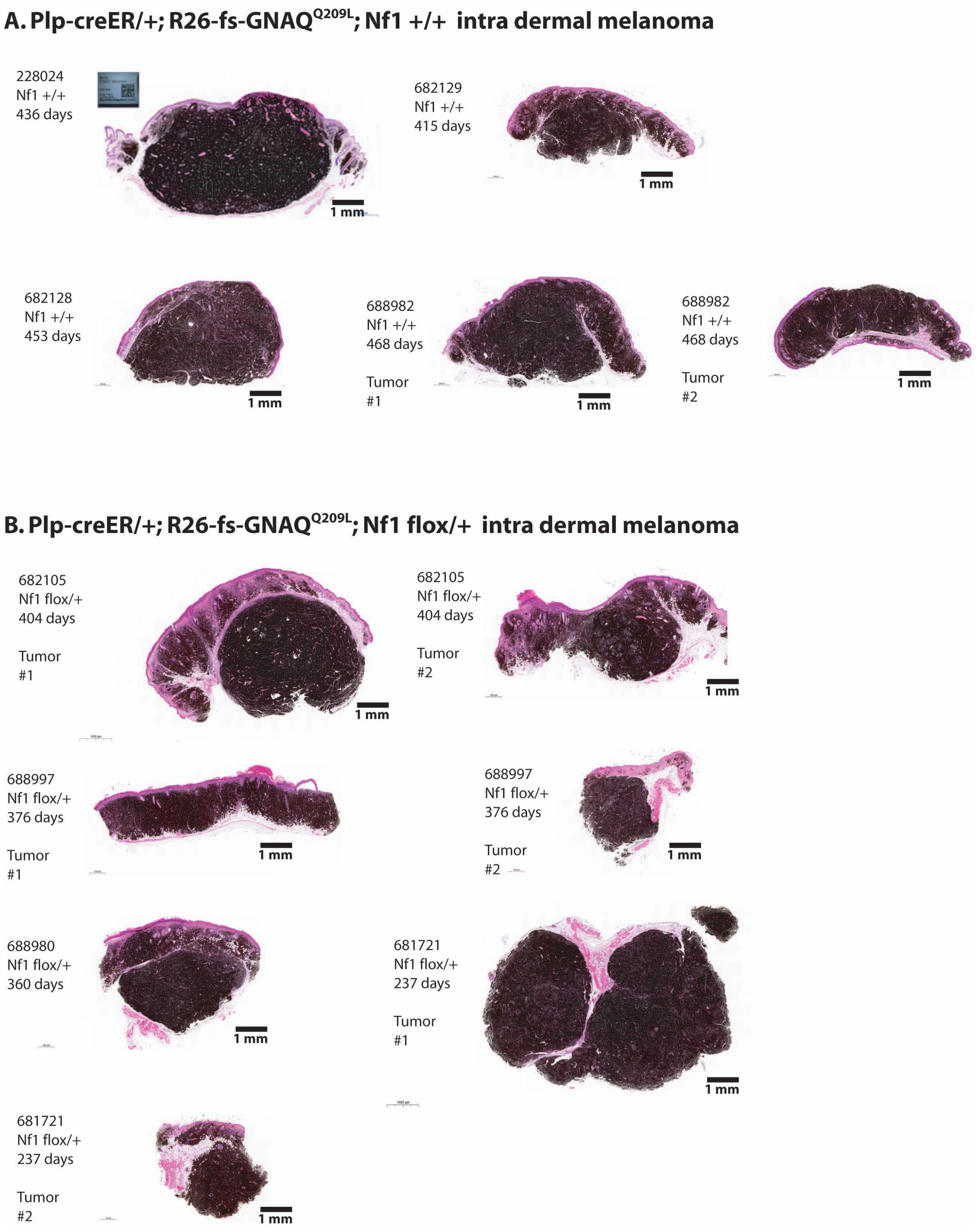

Supplementary Figure 2. H&E stained sections of all intra-dermal melanomas. (A,B) H&E stained sections of intra dermal melanomas isolated from *Plp1-creERT*/+; *R26-fs-GNAQ*^{Q209L}/+; +/+ (A) and *Plp1-creERT*/+; *R26-fs-GNAQ*^{Q209L}/+; *Nf1*^{flox}/+ (B) mice injected with tamoxifen at 5 weeks of age. To the left of each image is further information (mouse ID number, tumor number if more than one, *Nf1* genotype, and days of survival after tamoxifen injection.)
