# Supplementary material for: Loss of NF1 Accelerates Uveal and Intradermal Melanoma Tumorigenesis, and Oncogenic GNAQ Transforms Schwann Cells
Source: Cancer Res Commun. 2025 Feb 3;5(2):209–25. doi: 10.1158/2767-9764.CRC-24-0386 (PMC11788999; doi:10.1158/2767-9764.CRC-24-0386)
Supplement: Supplementary Figure 3 [file crc-24-0386_supplementary_figure_3_suppsf3.pdf]

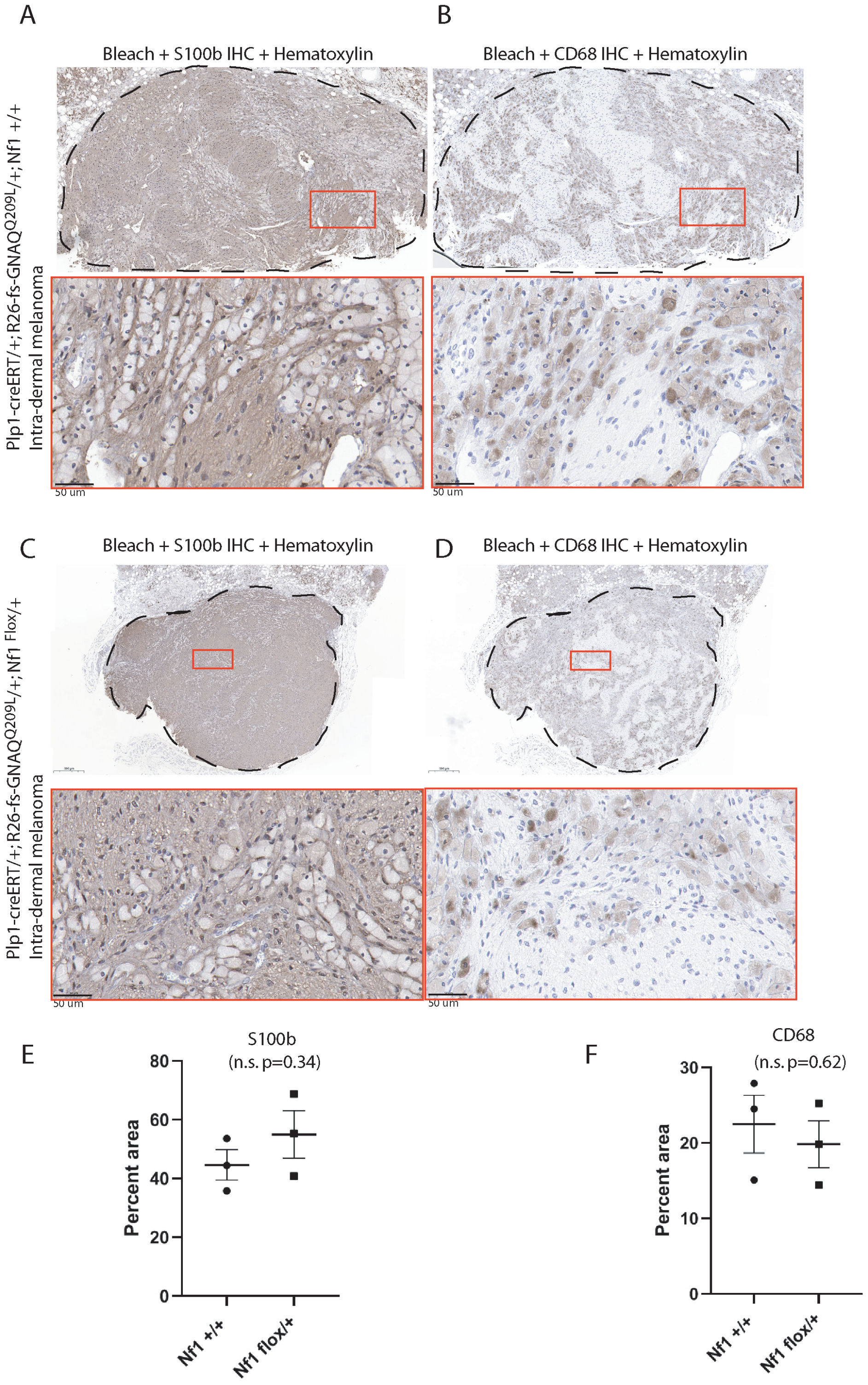

Supplementary Figure 3. Histological analysis of intra-dermal melanomas. (A-D) Representative images of S100b IHC (A,C) or CD68 (B,D) on intra-dermal melanomas from *Plp1-creERT*/+; *R26-fs-GNAQ*^{Q209L}/+; +/+ (A,B) and *Plp1-creERT*/+; *R26-fs-GNAQ*^{Q209L}/+; *Nf1*^{flox}/+ (C,D) mice injected with tamoxifen at 5 weeks of age. Sections were bleached of melanin before antibody staining and counterstained with haematoxylin. In both tumors, there are a large number of CD68 positive cells, which have the cellular morphology of macrophages/melanophages (large, round, and prior to bleaching, pigmented). Note the complimentary patterns of S100b-positivity and CD68-positivity in the serial sections. **(E,F)** Quantification of S100b (E) and CD68 (F) signal in *Plp1-creERT*/+; *R26-fs-GNAQ*^{Q209L}/+; +/+ versus *Plp1-creERT*/+; *R26-fs-GNAQ*^{Q209L}/+; *Nf1*^{flox}/+ intra-dermal melanoma. The percent area of the tumor that was positive for each sample is graphed. There was no significant difference found (p= 0.34 and p=0.62 respectively; unpaired t test). Error bars represent standard error of the mean.
