# Supplementary material for: Loss of NF1 Accelerates Uveal and Intradermal Melanoma Tumorigenesis, and Oncogenic GNAQ Transforms Schwann Cells
Source: Cancer Res Commun. 2025 Feb 3;5(2):209–25. doi: 10.1158/2767-9764.CRC-24-0386 (PMC11788999; doi:10.1158/2767-9764.CRC-24-0386)
Supplement: Supplementary Figure 4 [file crc-24-0386_supplementary_figure_4_suppsf4.pdf]

A. Plp-creER/+; R26-fs-GNAQ^{Q209L}; Nf1 +/+ mice with sparse pigment tumors

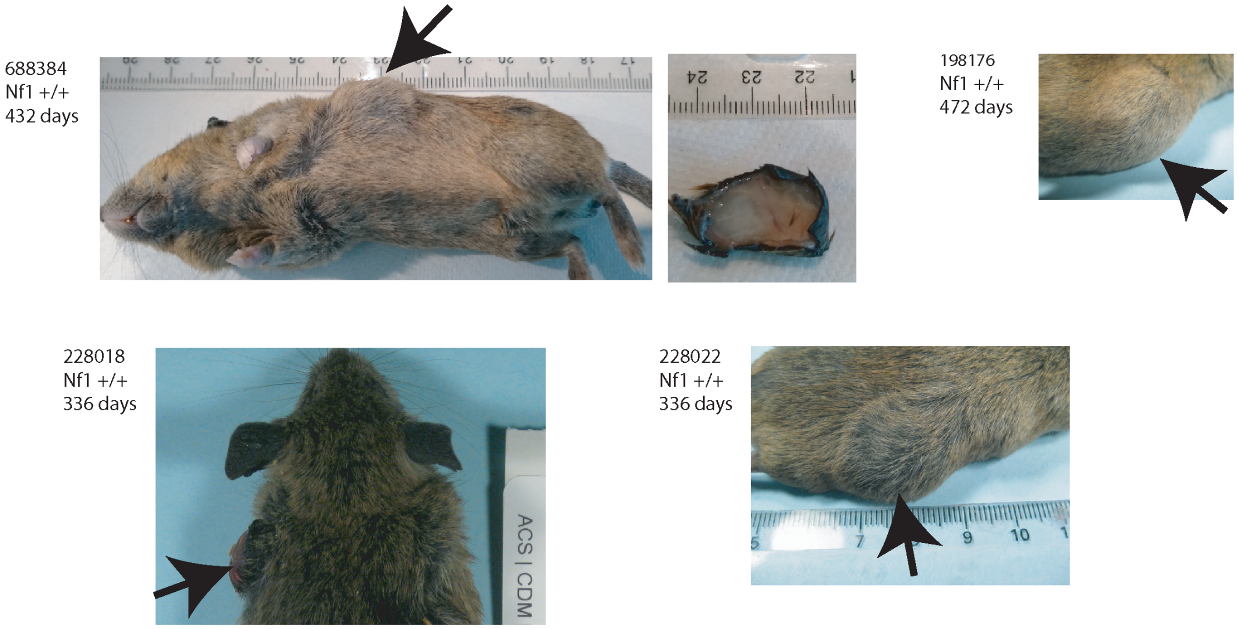

B. Plp-creER/+; R26-fs-GNAQ^{Q209L}; Nf1 flox/+ mice with sparse pigment tumors

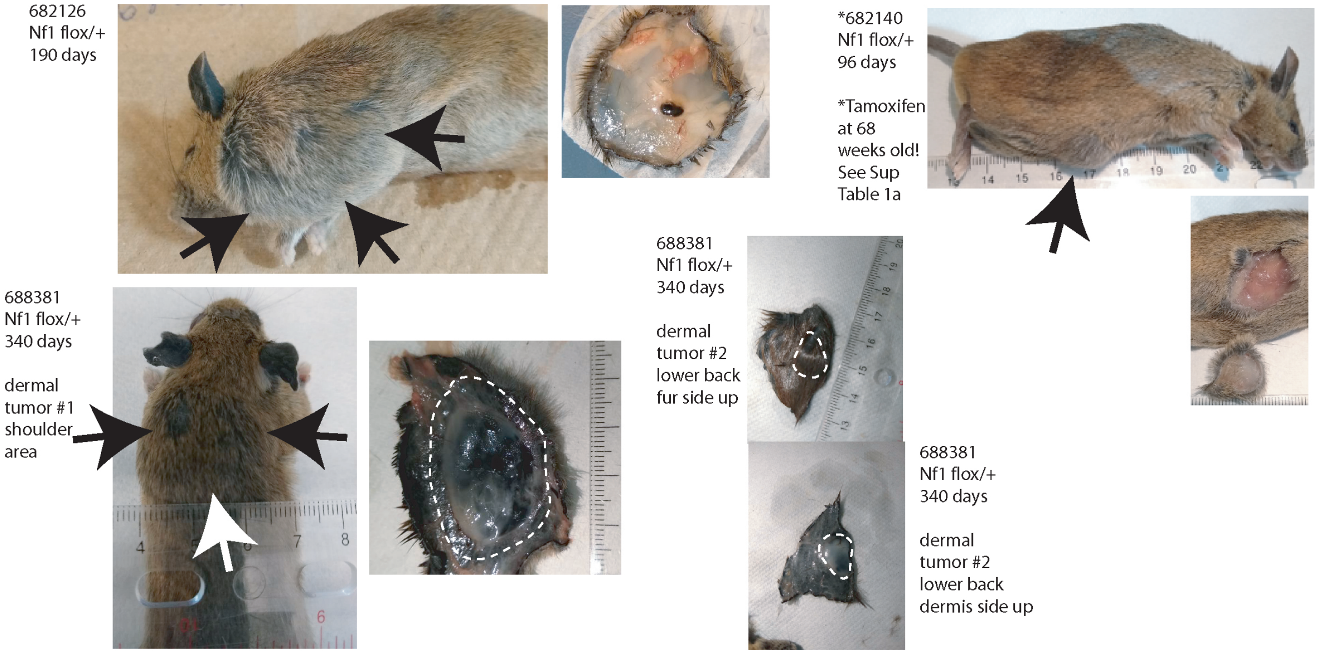

Supplementary Figure 4. Necroscopy photos of all sparse pigment tumors in the dermis. (A,B) Photos of *Plp1-creERT*/+; *R26-fs-GNAQ*^{Q209L}/+; +/+ (A) and *Plp1-creERT*/+; *R26-fs-GNAQ*^{Q209L}/+; *Nf1*^{flox}/+ (B) mice with sparse pigment tumors in the dermis, some with the tumor dissected to the right. To the left of each image is further information (mouse ID number, tumor number if more than one, *Nf1* genotype, and days of survival after tamoxifen injection.) Tumors are indicated with white or black arrows, or white dotted lines.
