# Supplementary material for: Loss of NF1 Accelerates Uveal and Intradermal Melanoma Tumorigenesis, and Oncogenic GNAQ Transforms Schwann Cells
Source: Cancer Res Commun. 2025 Feb 3;5(2):209–25. doi: 10.1158/2767-9764.CRC-24-0386 (PMC11788999; doi:10.1158/2767-9764.CRC-24-0386)
Supplement: Supplementary Figure 6 [file crc-24-0386_supplementary_figure_6_suppsf6.pdf]

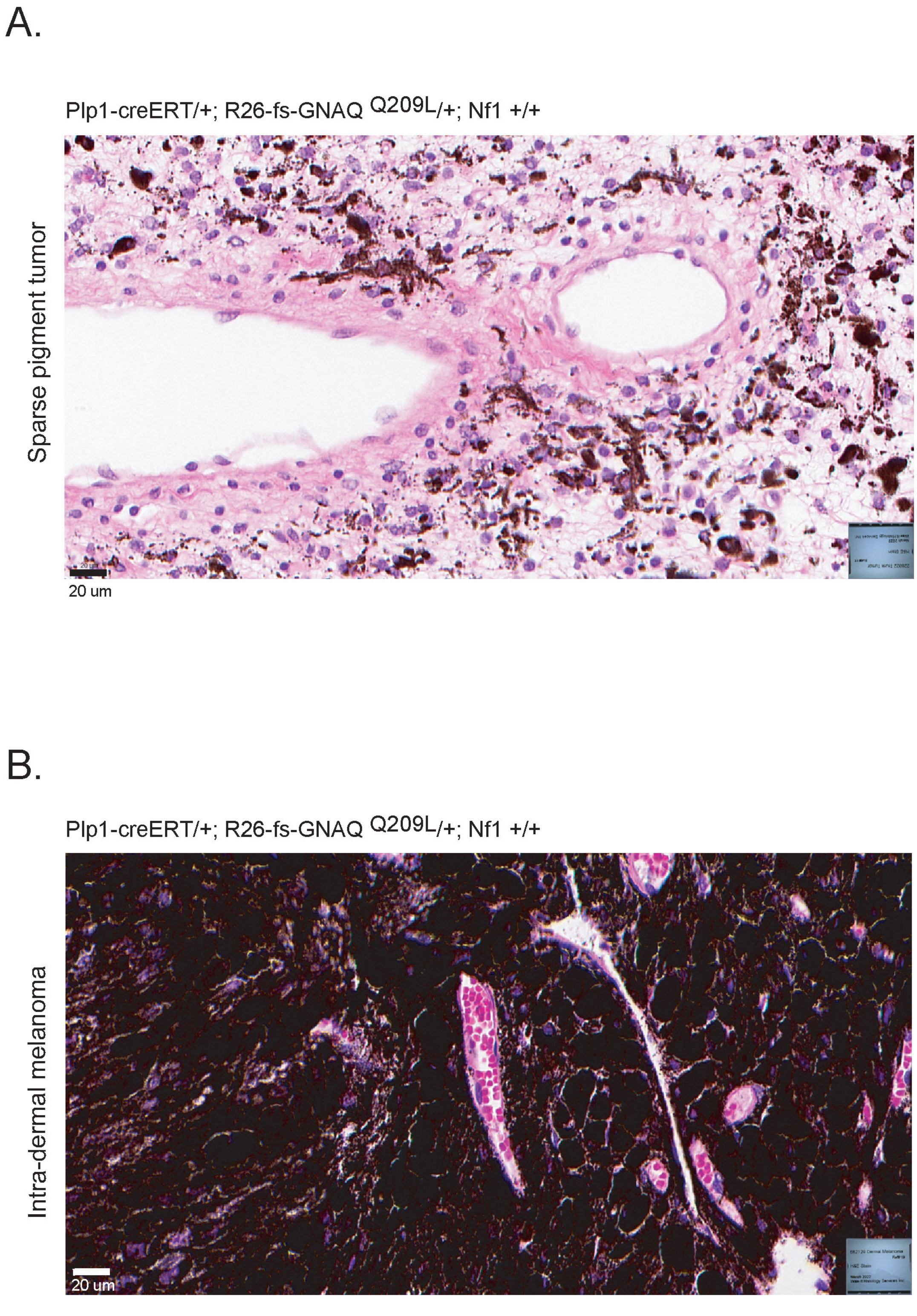


**Supplementary Figure 6. Comparison of blood vessels in a sparse pigment tumor and an intra-dermal melanoma from *Plp1-creERT*/+; *R26-fs-GNAQ*$^{Q209L}$/+; +/+ mice. (A,B)** H&E stained sections of a sparse pigment tumor (A) and an intra-dermal melanoma (B). The vessel walls of the sparse pigment tumor are thicker and stained brighter pink with eosin.
