# Supplementary material for: Loss of NF1 Accelerates Uveal and Intradermal Melanoma Tumorigenesis, and Oncogenic GNAQ Transforms Schwann Cells
Source: Cancer Res Commun. 2025 Feb 3;5(2):209–25. doi: 10.1158/2767-9764.CRC-24-0386 (PMC11788999; doi:10.1158/2767-9764.CRC-24-0386)
Supplement: Supplementary Figure 7 [file crc-24-0386_supplementary_figure_7_suppsf7.pdf]

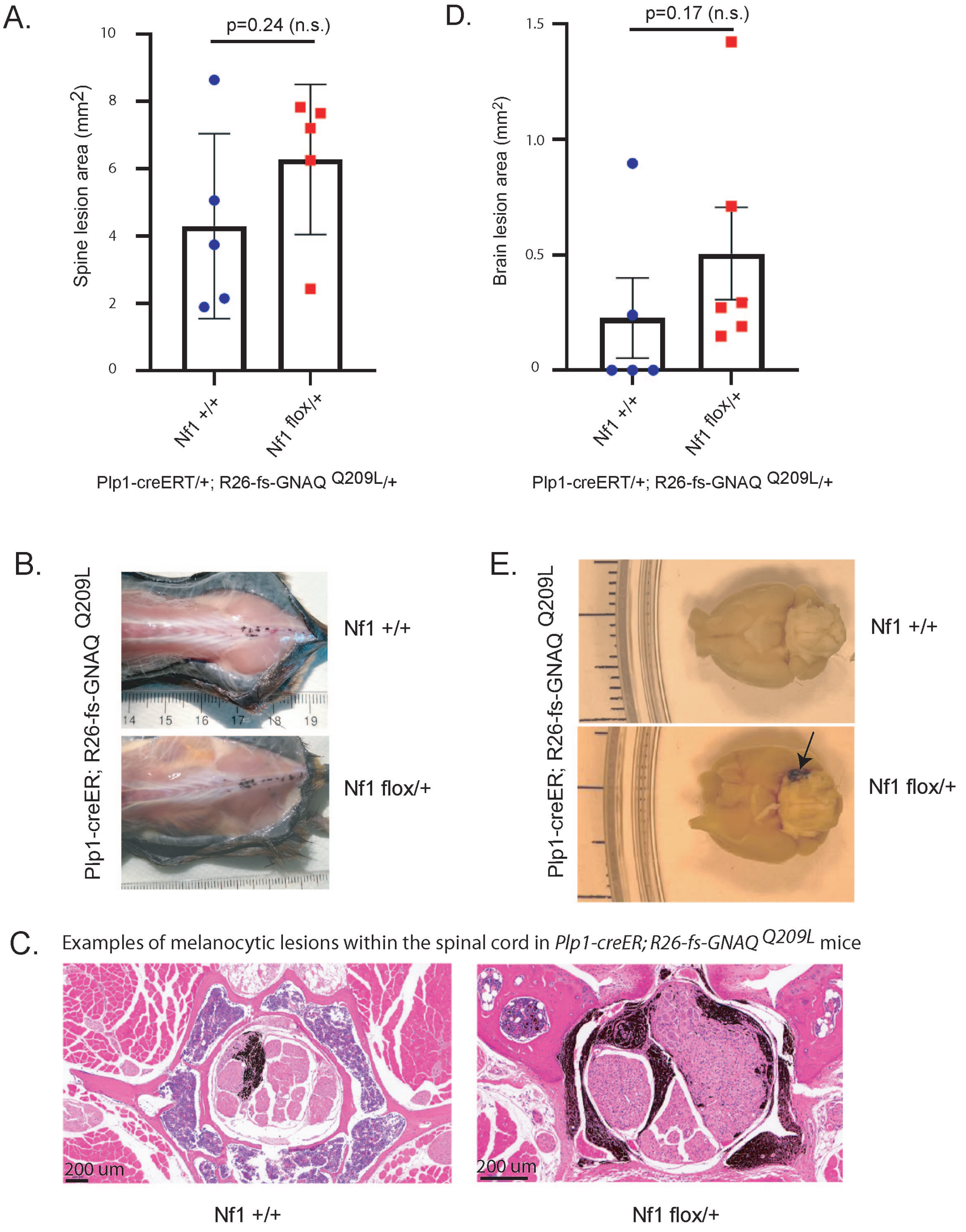

Supplementary Figure 7. There was no significant difference found in CNS phenotypes between *Nf1* genotypes. (A) Graph showing average area of pigmented lesions associated with the spine in *Plp1-creERT*/+; *R26-fs-GNAQ*^{Q209L}/+; *Nf1*^{flox}/+ and *Plp1-creERT*/+; *R26-fs-GNAQ*^{Q209L}/+; +/+ mice injected with tamoxifen at 5 weeks of age and euthanized upon tumor or other humane endpoint. **(B)** Representative *Plp1-creERT*/+; *R26-fs-GNAQ*^{Q209L}/+; *Nf1*^{flox}/+ and *Plp1-creERT*/+; *R26-fs-GNAQ*^{Q209L}/+; +/+ mice exhibiting darkly pigmented lesions associated with the spine. **(C)** H&E stained sections of spines showing melanocytic growth within neural tissues. **(D)** Graph showing average area of pigmented lesions associated with the ventral brain surface in *Plp1-creERT*/+; *R26-fs-GNAQ*^{Q209L}/+; *Nf1*^{flox}/+ and *Plp1-creERT*/+; *R26-fs-GNAQ*^{Q209L}/+; +/+ mice. **(E)** Representative *Plp1-creERT*/+; *R26-fs-GNAQ*^{Q209L}/+; *Nf1*^{flox}/+ and *Plp1-creERT*/+; *R26-fs-GNAQ*^{Q209L}/+; +/+ brains, ventral side up. Arrow indicates a pigmented lesion. Although there was no significant difference, the trends suggest that with more animals, *Nf1*^{flox}/+ might cause greater growth of CNS associated lesions. Error bars in graphs represent the standard error of the mean (SEM).
