# Supplementary material for: Loss of NF1 Accelerates Uveal and Intradermal Melanoma Tumorigenesis, and Oncogenic GNAQ Transforms Schwann Cells
Source: Cancer Res Commun. 2025 Feb 3;5(2):209–25. doi: 10.1158/2767-9764.CRC-24-0386 (PMC11788999; doi:10.1158/2767-9764.CRC-24-0386)
Supplement: Supplementary Figure 8 [file crc-24-0386_supplementary_figure_8_suppsf8.pdf]

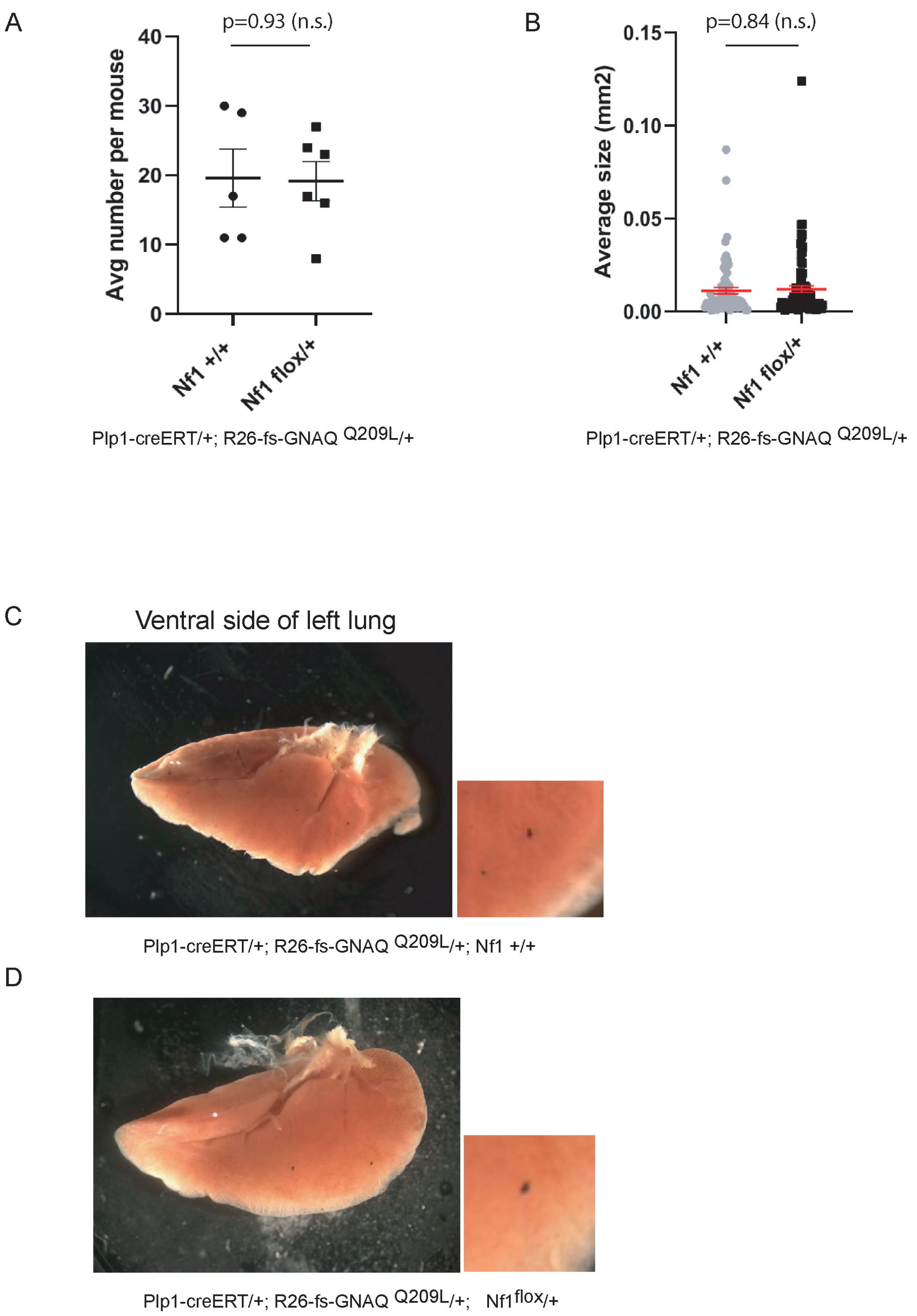

Supplementary Figure 8. There was no significant difference in lung phenotypes between *Nf1* genotypes. (A) Graph showing average number of pigmented lung lesions per mouse and (B) the average size of lesions (lesions pooled together) of the second cohort of mice: 5 *Plp1-creERT*/+; *R26-fs-GNAQ*^{Q209L}/+; +/+ and 6 *Plp1-creERT*/+; *R26-fs-GNAQ*^{Q209L}/+; *Nf1*^{flox}/+ mice injected with tamoxifen at 5 weeks of age and euthanized upon tumor or other humane endpoint. There was no significant difference in either measurement (student t test and Mann-Whitney test, respectively). Error bars in graphs represent the standard error of the mean (SEM). **(C,D)** Representative left lungs from *Plp1-creERT*/+; *R26-fs-GNAQ*^{Q209L}/+; +/+ (C) and *Plp1-creERT*/+; *R26-fs-GNAQ*^{Q209L}/+; *Nf1*^{flox}/+ (D) mice.
