# Supplementary material for: Loss of NF1 Accelerates Uveal and Intradermal Melanoma Tumorigenesis, and Oncogenic GNAQ Transforms Schwann Cells
Source: Cancer Res Commun. 2025 Feb 3;5(2):209–25. doi: 10.1158/2767-9764.CRC-24-0386 (PMC11788999; doi:10.1158/2767-9764.CRC-24-0386)
Supplement: Supplementary Figure 9 [file crc-24-0386_supplementary_figure_9_suppsf9.pdf]

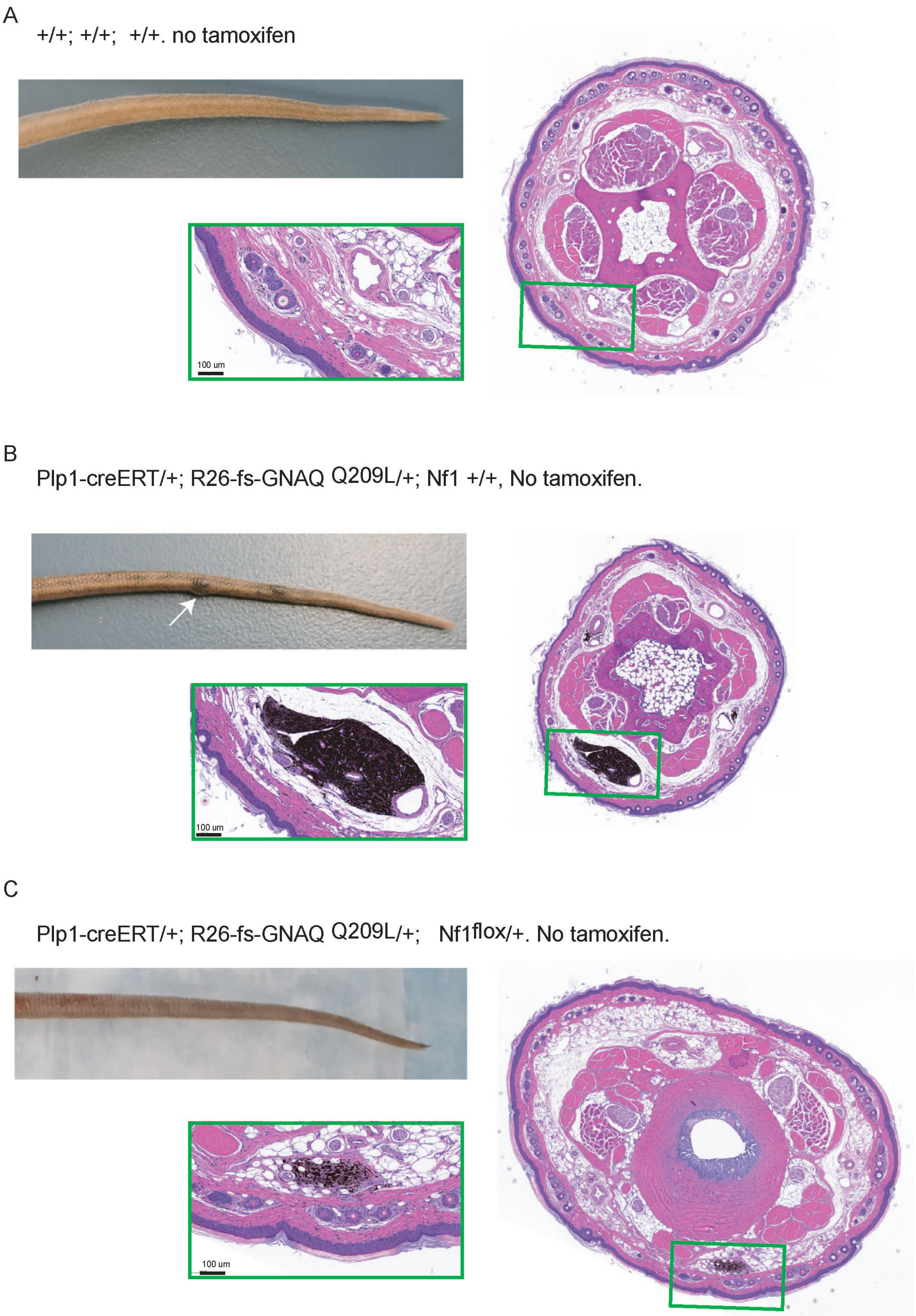

Supplementary Figure 9. Tail phenotypes in control mice not injected with tamoxifen. (A,B,C). Whole tails and H&E stained sections of +/+; +/+; +/+ (A), *Plp1-creERT*/+; *R26-fs-GNAQ*^{Q209L}/+; +/+ (B), or *Plp1-creERT*/+; *R26-fs-GNAQ*^{Q209L}/+; *Nf1*^{flox}/+ (C) mice that were housed in tamoxifen-free cages. Two cases of tail lesions that were found are shown in B and C, indicating that there is some small amount of leaky CreERT activity. Animals were healthy and aged to 72 weeks.
