# Supplementary material for: Loss of NF1 Accelerates Uveal and Intradermal Melanoma Tumorigenesis, and Oncogenic GNAQ Transforms Schwann Cells
Source: Cancer Res Commun. 2025 Feb 3;5(2):209–25. doi: 10.1158/2767-9764.CRC-24-0386 (PMC11788999; doi:10.1158/2767-9764.CRC-24-0386)
Supplement: Supplementary Figure 10 [file crc-24-0386_supplementary_figure_10_suppsf10.pdf]

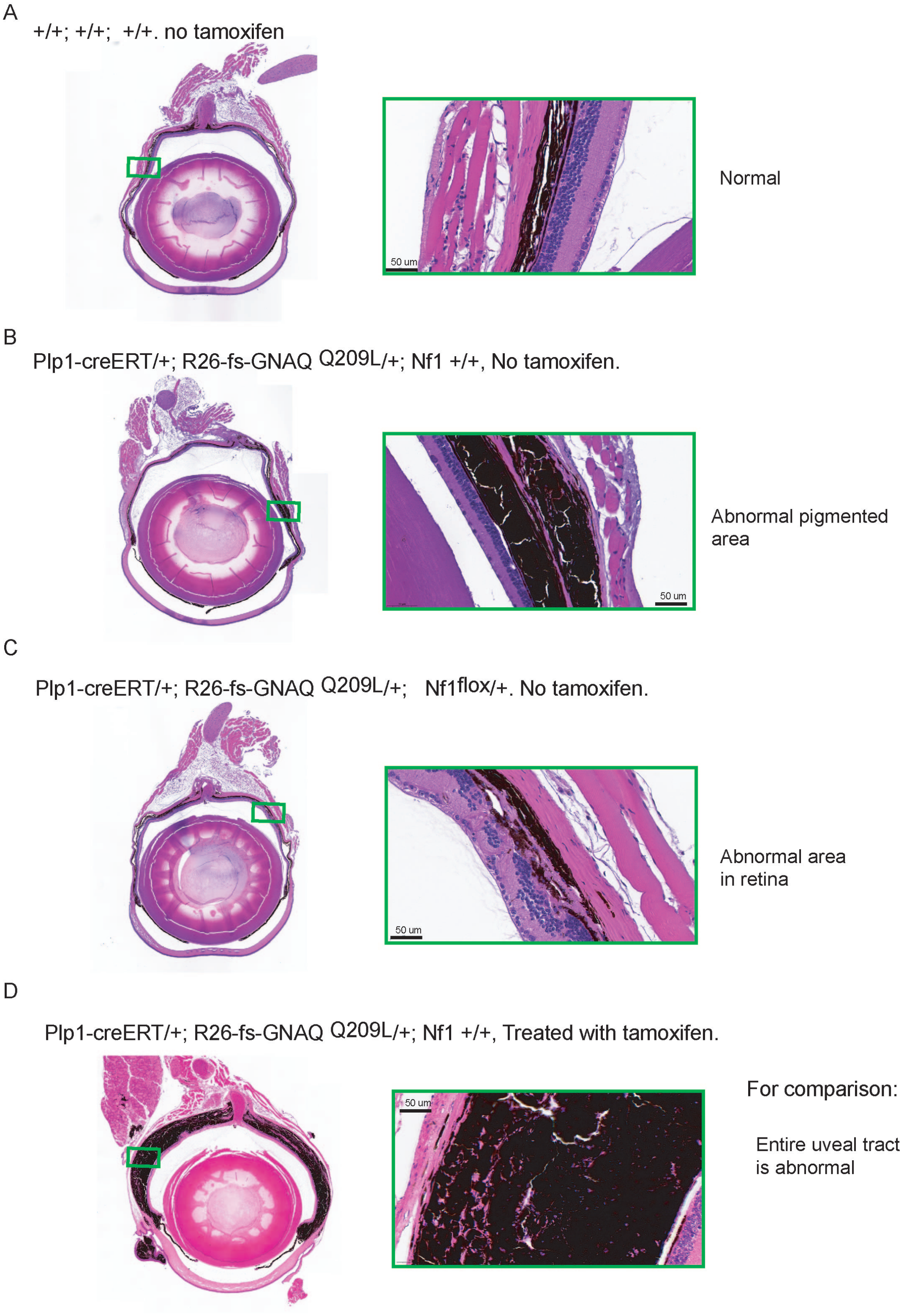

Supplementary Figure 10. Eye phenotypes in control mice not injected with tamoxifen. (A,B,C). Eye sections stained with H&E from +/+; +/+; +/+ (A), *Plp1-creERT*/+; *R26-fs-GNAQ*^{Q209L}/+; +/+ (B), or *Plp1-creERT*/+; *R26-fs-GNAQ*^{Q209L}/+; *Nf1*^{flox}/+ (C) mice that were housed in tamoxifen-free cages until 72 weeks old. One eye was found with a small area of expanded pigmentation (boxed area in B), which suggests that there is some small amount of leaky CreERT activity in ocular melanocytes. Another eye exhibited a disorganized neural retina, of unknown significance to the study (boxed area in C). **(D)** For reference, a tamoxifen treated *Plp1-creERT*/+; *R26-fs-GNAQ*^{Q209L}/+; +/+ mouse eye shows a much more enhanced growth of the uveal tract.
