# Supplementary material for: Loss of NF1 Accelerates Uveal and Intradermal Melanoma Tumorigenesis, and Oncogenic GNAQ Transforms Schwann Cells
Source: Cancer Res Commun. 2025 Feb 3;5(2):209–25. doi: 10.1158/2767-9764.CRC-24-0386 (PMC11788999; doi:10.1158/2767-9764.CRC-24-0386)
Supplement: Supplementary Figure 11 [file crc-24-0386_supplementary_figure_11_suppsf11.pdf]

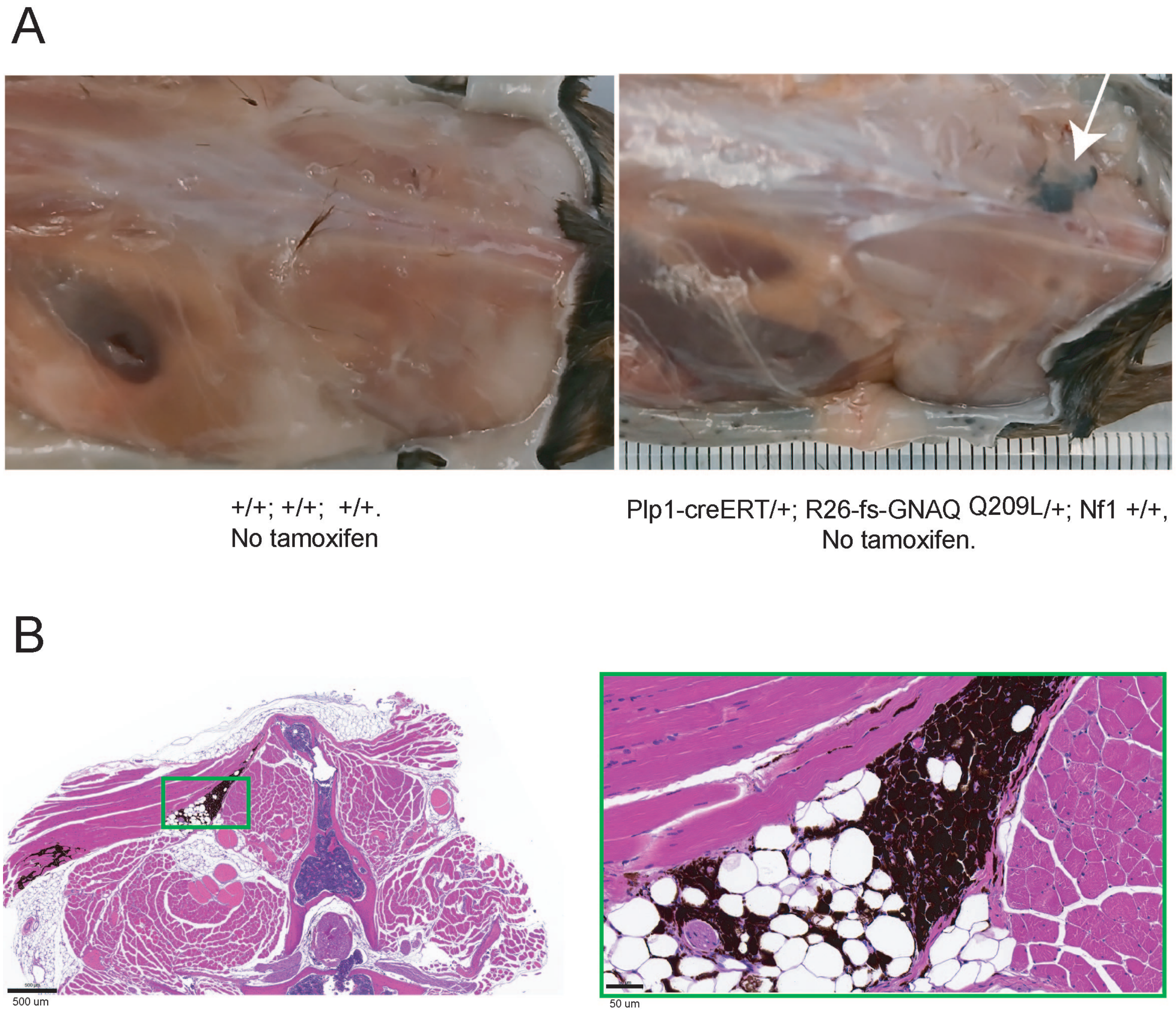

Supplementary Figure 11. Spine phenotypes in control mice not injected with tamoxifen. (A). Macroscopic image of the spine of a +/+; +/+; +/+ (left) and *Plp1-creERT*/+; *R26-fs-GNAQ*^{Q209L}/+; +/+ (right) mouse housed in tamoxifen-free cages until 72 weeks old. The *Plp1-creERT*/+; *R26-fs-GNAQ*^{Q209L}/+; +/+ mouse exhibited a pigmented lesion, indicated with the arrow. **(B)** H&E stained section through the lesion shown in A. Invasion of the muscle layer is apparent. This lesion suggests that there is some small amount of leaky CreERT activity in melanocytes associated with the CNS and normally found in the meninges.
