# Supplementary material for: Loss of NF1 Accelerates Uveal and Intradermal Melanoma Tumorigenesis, and Oncogenic GNAQ Transforms Schwann Cells
Source: Cancer Res Commun. 2025 Feb 3;5(2):209–25. doi: 10.1158/2767-9764.CRC-24-0386 (PMC11788999; doi:10.1158/2767-9764.CRC-24-0386)
Supplement: Supplementary Figure 12 [file crc-24-0386_supplementary_figure_12_suppsf12.pdf]

A

Intra-dermal melanoma

genes up or down in *Nf1 flox/+* tumors

DE genes:

Down	Up	
Dclk1	*2610035D17Rik*	*Rpl26*
Fosb	***Adcy1***	*S100b*
Gm27177	***Atp1b2***	*Scn2b*
Gm28036	*Cmtm5*	*Slc40a1*
Habp2	*Eps8l2*	***Tceal3***
Pak3	*Fcrls*	
Pde10a	***Gfra2***	
Pde3a	*Gzmc*	
Ppm1l	*Ntn4*	
Sall4	*Rcan2*	

B

Uveal melanoma

genes up or down in *Nf1 flox/+* tumors

DE genes:

Down			Up
Acta1	*Egfl6*	*Tnni2*	***Foxp4***
Actn3	*Mybpc2*	*Tnnt3*	*Rpl26*
Atp2a1	*Myh4*	*Tpm2*	
Bhmt	*Mylk2*		
Bmx	*Myom2*		
Casq1	*Myoz1*		
Ckm	*Pgam2*		
Cmya5	*Phkg1*		
Cox6a2	*Pvalb*		
Dhrs7c	*Synpo2*		

C **Down regulated genes in *Nf1* flox/+ tumors correlated with survival in human UM**

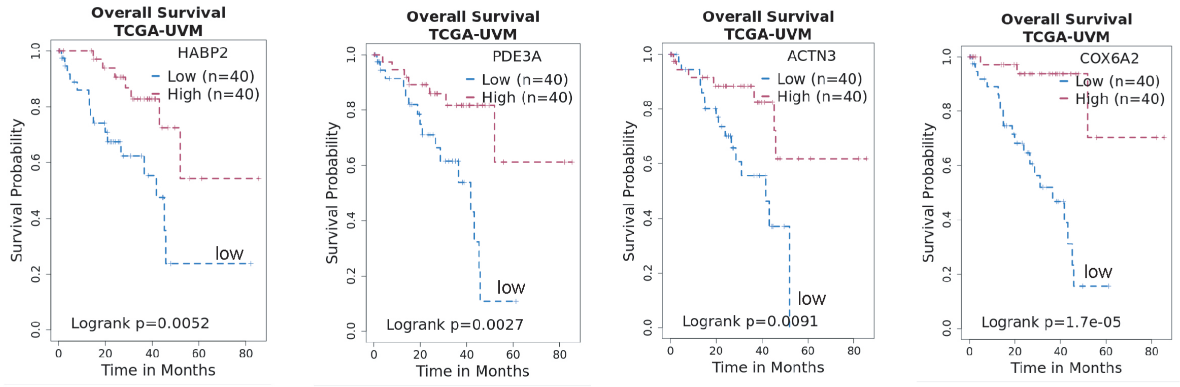

D **Up regulated genes in *Nf1* flox/+ tumors correlated with survival in human UM**

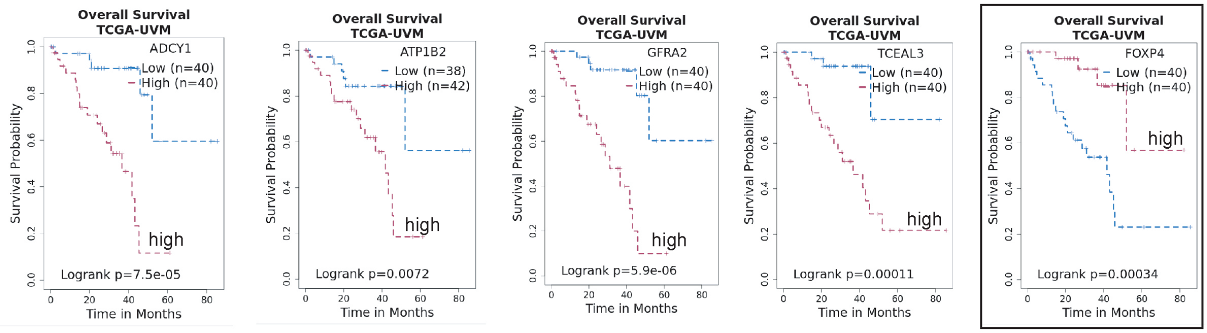

* opposite correlation

Supplementary Figure 12. A subset of differentially expressed genes in *Nf1* mutant mouse melanomas is associated with survival in human uveal melanomas. A,B) List of all significant up- and down-regulated genes in $Nf1^{flox}$/+ intra-dermal (A) or uveal (B) melanoma ($p_{adj} < 0.05$)**.** Bolded genes are associated with survival in human uveal melanoma. **C,D)** Kaplan-Meier survival plots of down-regulated (C) and up-regulated (D) differentially expressed genes that were significantly associated with survival in the TCGA-UVM dataset. Among the genes that were correlated, genes whose low expression was associated with worse survival were down-regulated in $Nf1^{flox}$/+ tumors, except for one gene, *Foxp4*. Conversely, genes whose high expression was associated with worse survival were up-regulated in $Nf1^{flox}$/+ tumors.
